# ^1^H-NMR as a Structural and Analytical Tool of Intra- and Intermolecular Hydrogen Bonds of Phenol-Containing Natural Products and Model Compounds

**DOI:** 10.3390/molecules190913643

**Published:** 2014-09-02

**Authors:** Pantelis Charisiadis, Vassiliki G. Kontogianni, Constantinos G. Tsiafoulis, Andreas G. Tzakos, Michael Siskos, Ioannis P. Gerothanassis

**Affiliations:** 1 Section of Organic Chemistry & Biochemistry, Department of Chemistry, University of Ioannina, Ioannina GR-45110, Greece; E-Mails: pcharis@cc.uoi.gr (P.C.); vkontog@cc.uoi.gr (V.G.K.); atzakos@cc.uoi.gr (A.G.T.); msiskos@cc.uoi.gr (M.S.); 2 NMR Center, University of Ioannina, Ioannina GR-45110, Greece; E-Mail: ctsiafou@cc.uoi.gr;

**Keywords:** chemical shifts, hydrogen bonding, ^1^H-NMR, ^1^H-^13^C HMBC, phenol OH, natural products

## Abstract

Experimental parameters that influence the resolution of ^1^H-NMR phenol OH signals are critically evaluated with emphasis on the effects of pH, temperature and nature of the solvents. Extremely sharp peaks (Δν_1/2_ ≤ 2 Hz) can be obtained under optimized experimental conditions which allow the application of ^1^H-^13^C HMBC-NMR experiments to reveal long range coupling constants of hydroxyl protons and, thus, to provide unequivocal assignment of the OH signals even in cases of complex polyphenol natural products. Intramolecular and intermolecular hydrogen bonds have a very significant effect on ^1^H OH chemical shifts which cover a region from 4.5 up to 19 ppm. Solvent effects on –OH proton chemical shifts, temperature coefficients (Δ*δ*/ΔT), OH diffusion coefficients, and *^n^J*(^13^C, O^1^H) coupling constants are evaluated as indicators of hydrogen bonding and solvation state of phenol –OH groups. Accurate ^1^H chemical shifts of the OH groups can be calculated using a combination of DFT and discrete solute-solvent hydrogen bond interaction at relatively inexpensive levels of theory, namely, DFT/B3LYP/6-311++G (2d,p). Excellent correlations between experimental ^1^H chemical shifts and those calculated at the *ab initio* level can provide a method of primary interest in order to obtain structural and conformational description of solute-solvent interactions at a molecular level. The use of the high resolution phenol hydroxyl group ^1^H-NMR spectral region provides a general method for the analysis of complex plant extracts without the need for the isolation of the individual components.

## 1. Introduction

Hydrogen bonding is a fundamental aspect of chemical structure, conformation and reactivity [1,2,3,4]. Detection of hydrogen bonds, therefore, remains an area of active research. NMR spectroscopy is one of the most powerful methods for investigating hydrogen bonding interactions both in solution and in the solid. Thus, ^1^H and heteronuclear chemical shifts, coupling constants, solvent and deuterium isotope effects on chemical shifts can provide evidence of hydrogen bonding [5]. Chemical shifts are also very sensitive to steric and electronic effects and to secondary and tertiary structure effects of biological macromolecules [6].

The use of hydroxyl protons in hydrogen bonding and conformational NMR studies in solution, presents experimental challenges due to rapid chemical exchange between hydroxyl groups and protic solvents. Proton exchange rates in alcohol –OH groups can be reduced by dissolving in DMSO-*d*_6_ or acetone-*d*_6_ [7], by supercooling aqueous solutions [8] or by using organic co-solvents [9] and, thus, have already been utilized in structural analysis of carbohydrates [9,10]. In contrast, the phenol –OH groups have rarely been investigated despite the fact that phenols are major constituents of many biological and naturally occurring compounds and phenols are almost ubiquitous in the plant and animal kingdom. Interestingly, around 7% of the current drugs contain phenol –OH.

Shapetko and Shigorin [11] provided a brief account of ^1^H-NMR studies of intramolecular hydrogen bonds of derivatives of hydroxynaphthoquinones, hydroxyanthraquinones, and tropolone which are an important class of natural products. Hansen and Spanget-Larsen [12] provided a comprehensive overview of NMR and IR studies of phenols both in solution and in the solid. In the present review article we will summarize ^1^H-NMR experimental parameters that influence the resolution of phenol –OH groups and provide an overview of recent developments in the use of ^1^H-NMR as structural, conformational and analytical tool of intra- and inter-molecular hydrogen bonds of phenol containing natural products and model compounds. 

## 2. Parameters Influencing Phenol –OH Proton Exchange Rates

The ^1^H-NMR resonances of phenol –OH groups display broad signals at room temperature due to intermolecular exchange of the –OH protons with protons of the protic solvents or with protons of the residual H_2_O in aprotic solvents. Further exchange broadening may be attributed to proton exchange between various –OH groups and –OH and –COOH groups due to intermolecular association of the solute molecules, particularly in low polarity and dielectric constant organic solvents. The phenol OH linewidths, therefore, are of vital importance in the assignment and interpretation of the ^1^H-NMR spectra. In the following sections the major factors influencing the proton exchange rates and, thus, OH linewidths will be evaluated.

### 2.1. Effects of pH

**Figure 1:**
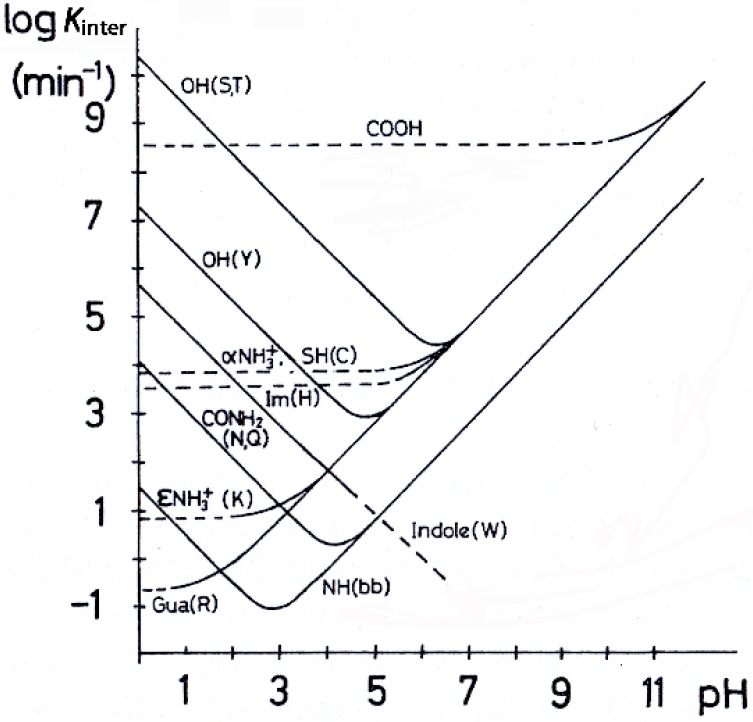
Effect of pH on ln*k*_inter_, where *k*_inter_ is the intermolecular proton exchange rate, for various functional groups in peptides and proteins. Adapted with permission from [15]. Copyright 1986, by John Wiley & Sons.

The chemical exchange of –OH and –NH protons in aqueous solution proceeds by way of distinct chemical reactions that may involve, in the role of catalysts, H_3_O^+^ and OH^−^, and general acids and bases including H_2_O [13,14,15]. A V-shaped curve was obtained experimentally for ln*k*_inter_, where *k*_inter_ is the proton intermolecular exchange rate, vs pH values [15] (Figure 1). For phenol –OH groups of tyrosine (Y) in peptides and proteins a minimum in aqueous solutions at pH 4.5 to 5.0 was obtained. For –OH groups of serine (S) and threonine (T) residues the minimum occurs in the region of pH 6.0 to 6.5. A minimum in the exchange rate of –OH protons has also been observed in mixtures of H_2_O with organic solvents [14]. It may be concluded that at pH values below this minimum the exchange rate is acid-catalyzed while above this it is OH^−^-catalyzed.

**Figure 2:**
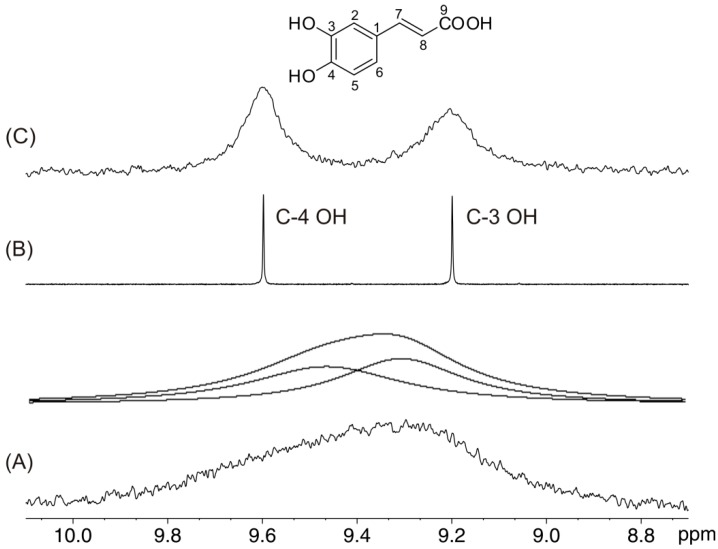
500 MHz 1D ^1^H-NMR spectra of the hydroxyl group region of caffeic acid (1) (C = 5.7 × 10^−3^ M, T = 292 K, number of scans = 32), in (**A**) DMSO-*d*_6_; upper trace: simulated line-shapes with two Lorentzian peaks for C-4 OH and C-3 OH having line-widths of 203 Hz and 164 Hz, respectively, using the Bruker peak-fitting routine; (**B**) and (**C**) with a molar ratio [picric acid]/[caffeic acid] of 12 × 10^−3^ and 219 × 10^−3^, respectively. Reproduced with permission from [16]. Copyright 2001, by the American Chemical Society.

Figure 2 illustrates the 1D ^1^H-NMR spectrum of caffeic acid (C = 5.7 × 10^−3^ M) in DMSO-*d*_6_ which exhibits extremely broad resonances of the C-4 OH (Δν_1/2_ = 203 Hz) and C-3 OH (Δν_1/2_ ≈ 164 Hz) groups. The addition of progressively increased amount from a solution of 2.1 × 10^−3^ M picric acid in DMSO-*d*_6_ resulted in a significant reduction in the C-4 OH and C-3 OH linewidths [16]. For a molar ratio [picric acid]/[caffeic acid] ≈ 12 × 10^−3^, a minimum in the linewidth of the C-4 OH (Δν_1/2_ ≈ 1.2 Hz) and C-3 OH (Δν_1/2_ ≈ 1.0 Hz) groups was obtained, with an average reduction in the linewidths by a factor of over 100. This allows the application of the two dimensional heteronuclear ^1^H–^13^C HMBC experiment for the complete assignment of the –OH groups (see discussion below).

Several comparative experiments were performed with the pH value adjusted for a minimum in the –OH proton exchange rate using acids that cover a range of p*K*_a_ values: HCl [17], CCl_3_COOH (p*K*_a_ = 0.66), CF_3_COOH (p*K*_a_ = 0.52), and picric acid (p*K*_a_ = 0.42). Picric acid was shown to be more effective in achieving line-widths in the range of 0.6 to 1.5 Hz. This result could be attributed to stacking modes of interaction of picric acid with aromatic systems [18] and hydrogen bonding interactions [19]. 

### 2.2. Effects of Temperature

**Figure 3:**
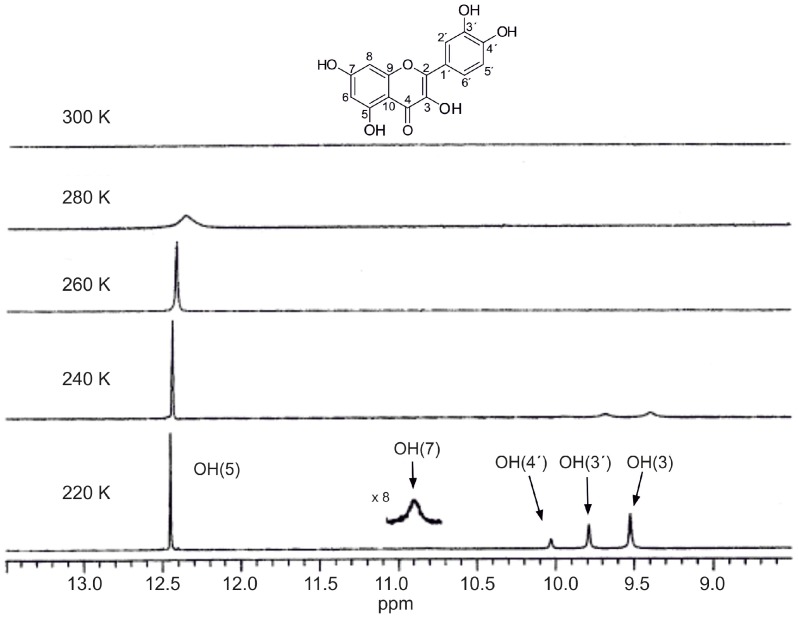
Variable temperature 400 MHz ^1^H-NMR spectra of quercetin in CD_3_OH, concentration 10 mM. Reproduced with permission from [20]. Copyright 2002, by Elsevier Science Ltd. (Amsterdam, The Netherlands).

Temperature has a profound effect on OH proton exchange rates. Generally in protic solvents the -OH groups appear at room temperature as broad signals due to fast, on the NMR time scale, exchange of the OH protons with protons of the solvents [20]. By decreasing the temperature, the proton exchange rate is reduced and relatively sharp –OH peaks are revealed. In the case of quercetin in methanol-*d*_3_ solution only a relatively broad OH resonance at ~12.3 ppm appears at 280 K whereas the other OH signals appear at temperatures < 240 K (Figure 3). The C-5 OH proton is the least mobile and accessible to the solvent, due to its participation in a strong intermolecular hydrogen bond between the C-5 OH and OC-4 group (see discussion below). 

Limbach *et al.* [21,22,23] investigated hydrogen bonded anions A•••H•••X^−^ of phenols (AH) and carboxylic/inorganic acids (HX). Very low temperatures were achieved (175 K in CDCl_3_ and 120 K in CDF_3_/CDF_2_Cl [24]) which allowed the investigation of individual hydrogen bonded species in the slow hydrogen bond exchange regime.

### 2.3. Effects of Solvents

**Figure 4:**
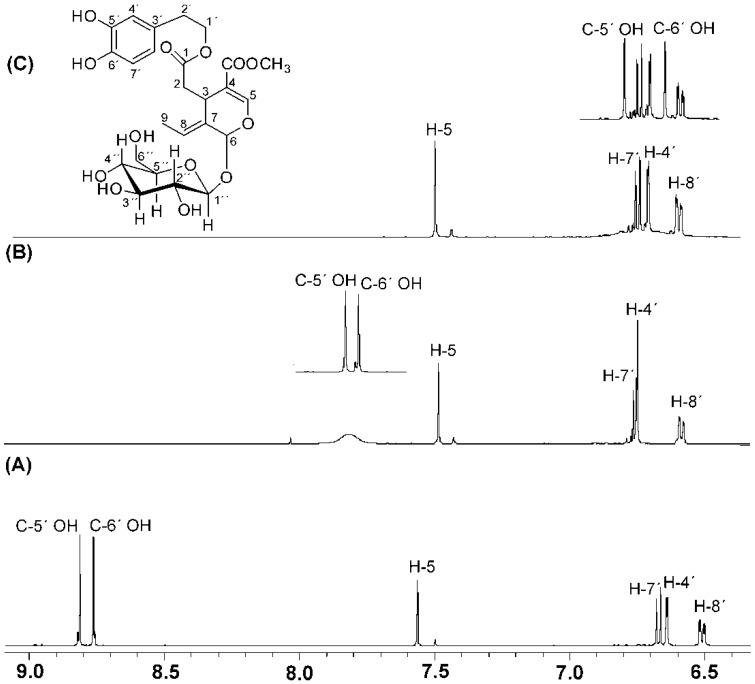
^1^H-NMR spectra (500 MHz) of oleuropein 6-*O*-*β*-d-glucopyranoside, concentration 5 mM (T = 292 K, number of scans = 64, experimental time = 8 min) in (**A**) DMSO-*d*_6_; (**B**) acetone-*d*_6_, upper trace after the addition of 2 μL picric acid, 8 mM in acetone-*d*_6_; and (**C**) CD_3_CN, upper trace after the addition of 2 μL picric acid, 8 mM in CD_3_CN. Reprinted with permission from [25]. Copyright 2013, The Royal Society of Chemistry.

Exchange broadening due to intermolecular proton exchange can be reduced with the use of dry non-protic solvents with strong hydrogen bonding ability. Figure 4 illustrates selected regions of the ^1^H-NMR spectra of oleuropein in three solvents which have varying degrees of hydrogen bonding and solvation abilities: DMSO-*d*_6_, acetone-*d*_6_ and CD_3_CN. In DMSO-*d*_6_ two very sharp resonances were observed for C-5′ OH (*δ* = 8.74 ppm, Δν_1/2_ ~ 1.6 Hz) and C-6′ OH (*δ* = 8.68 ppm, Δν_1/2_ ~ 1.7 Hz) [25]. In acetone-*d*_6_ the two resonances appear as a composite broad resonance (Δν_1/2_ ~ 35.9 Hz) which is shifted to low frequency (*δ* *≈* 7.74 ppm). In CD_3_CN, the composite signal, which is further shifted to low frequency in the aromatic region, cannot be distinguished from the baseline due to extensive broadening.

The use, therefore, of DMSO as solvent has three distinct advantages: (i) it reduces significantly proton exchange rates and, thus, OH linewidths due to its strong hydrogen bonding and solvation ability; (ii) the –OH resonances are well outside the overcrowded region of the aromatic protons and, thus, of high diagnostic value for the identification of the –OH resonances (see discussion below), and (iii) it is an excellent solvent for the majority of polyphenol compounds.

## 3. Assignments of Phenol –OH Resonances

**Figure 5:**
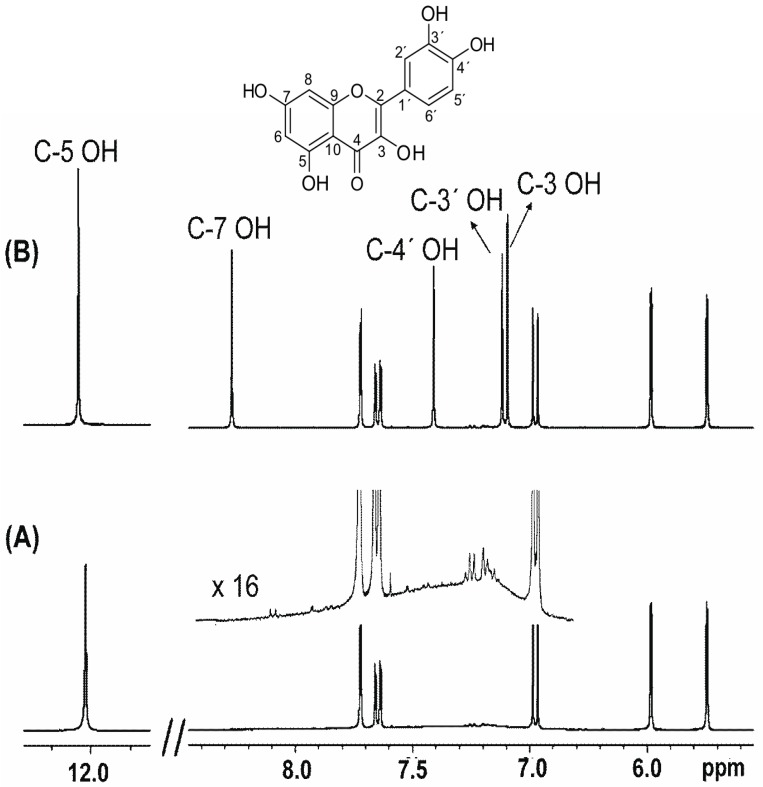
^1^H-NMR spectra (400 MHz) of quercetin, concentration 5 mM (T = 280 K, number of scans = 32, experimental time = 4 min) in (**A**) CD_3_CN, upper trace after vertical expansion (×16), and (**B**) the same solution as in (**A**) with the presence of 2 μL picric acid, 8 mM. Reprinted with permission from [25]. Copyright 2013, The Royal Society of Chemistry.

The 1D ^1^H-NMR spectrum of quercetin in CD_3_CN (at 280 K) exhibits a sharp and a strongly deshielded resonance at 12.05 ppm (Figure 5A) which is attributed to the C-5 OH proton of flavonoids. In contrast, the C-3, C-3′, and C-4′ OH resonances appear as extremely broad and unresolved resonances in the region of 7.0 to 7.5 ppm, while the C-7 OH group cannot be distinguished from the baseline. By decreasing the temperature the proton exchange is reduced, however, even at 240 K the resonances are still quite broad, which prohibits accurate determination of chemical shifts and investigation of solvent effects. Addition of 2 μL of picric acid (8 mM in CD_3_CN) results in extremely sharp resonances (Δν_1/2_ < 2.5 Hz) of all the phenol –OH resonances at 280 Κ (Figure 5B) [25]. This allowed the application of ^1^H–^13^C HMBC-NMR experiments to reveal long range coupling of hydroxyl protons and, thus, to provide unequivocal assignment of the –OH signals. Thus, the C-5 OH at 12.05 ppm shows cross peaks with carbons C-5 (161.6 ppm), C-6 (98.8 ppm) and C-10 (104.1 ppm), the C-7 OH at 8.29 ppm shows cross peaks with carbons C-7 (164.2 ppm), C-8 (94.7 ppm) and C-6 (98.8 ppm), and the C-3 OH at 7.11 ppm illustrates cross peaks with carbons C-3 (136.0 ppm) and C-4 (176.2 ppm). Cross peaks between C-4′ OH (7.43 ppm) with C-4′ (147.5 ppm), C-3′ (144.9 ppm) and C-5′ (116.0 ppm) and C-3′ OH (7.13 ppm) with C-3′ (144.9 ppm), C-4′ (147.5 ppm) and C-2′ (115.2 ppm) were also clearly observed.

**Figure 6:**
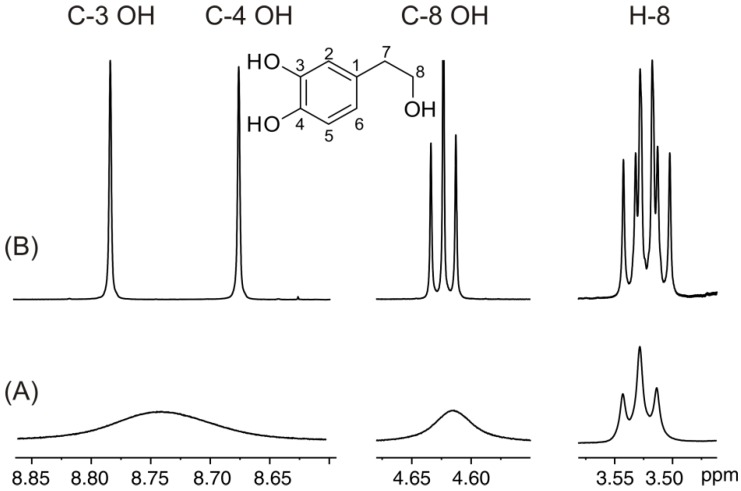
500 MHz 1D ^1^H-NMR spectra of hydroxytyrosol (C = 19.6 × 10^−3^ M, T = 288 K, number of scans = 16), in (**A**) DMSO-*d*_6_; (**B**) with a molar ratio of [picric acid]/[hydroxytyrosol] of 1.3 × 10^−3^. Reproduced with permission from [16]. Copyright 2011, by the American Chemical Society.

**Figure 7:**
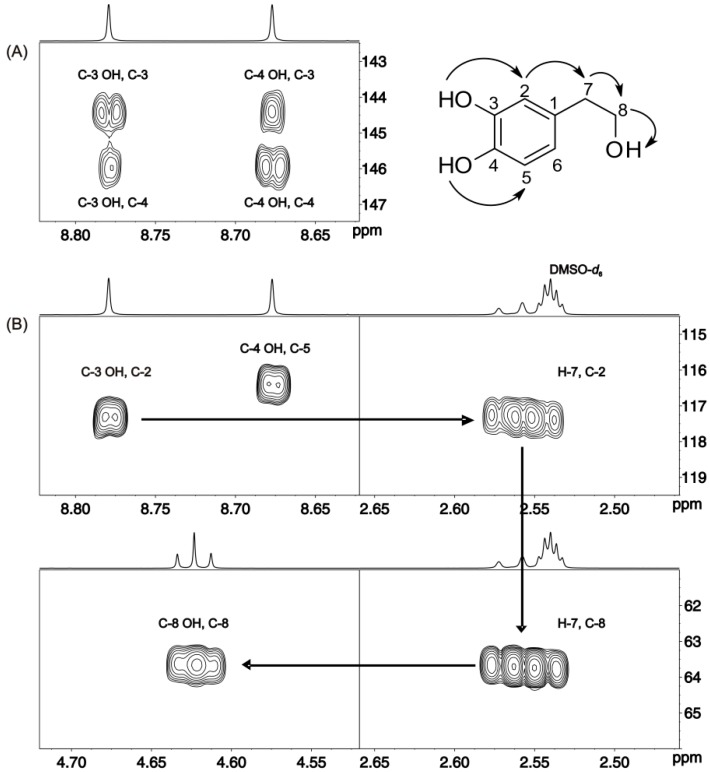
Selected regions of the 500 MHz 2D ^1^H-^13^C HMBC-NMR spectrum of the solution of Figure 6B (T = 288 K, number of scans = 8, experimental time = 1 h). The experiment was optimized for *^n^J* (^1^H, ^13^C) values for 6 to 8 Hz. Reproduced with permission from [16]. Copyright 2011, by the American Chemical Society.

The ^1^H-NMR spectrum of hydroxytyrosol (Figure 6A) illustrates a composite broad signal (Δν_1/2_ ≈ 48 Hz) of the C-3 OH and C-4 OH groups. Addition of picric acid resulted in a very significant reduction of the linewidths of the C-3 OH, C-4 OH, -CH_2_OH and the H-8 protons (Figure 6B). The extremely sharp –OH resonances (Δν_1/2_ ≈ 1.0 Hz) allowed the application of the 2D ^1^H-^13^C HMBC method and, thus, the complete assignment of ^1^H and ^13^C resonances. For example, the common *^2,3^J*(^1^H, ^13^C) cross-peaks of the C-4 and C-3 hydroxyl protons to carbons C-4 and C-3 indicated that these two OH groups are in an *ortho* position with respect to each other (Figure 7A). The *^3^J* (^1^H, ^13^C) cross-peak of the C-3 OH group to C-2 allows further connectivity to the H-7 proton and, thus, through ^2^*J*(^1^H, ^13^C) to the C-8 carbon and furthermore to the C-8 OH group (Figure 7B).

## 4. Phenol OH Proton Shieldings

### 4.1. –OH Shielding Range—Effects of Hydrogen Bonding

Spapetko and Shigorin provided a ^1^H-NMR investigation of strong intramolecular hydrogen bonds of quinoid compounds [11]. *δ*_ΟΗ_ values for six-membered and five-membered conjugated rings with intramolecular hydrogen bonds are significantly different (Table 1). This was explained by increases in bond lengths through stresses arising in the five-membered rings which result in weakening of the hydrogen bonds. When a six-membered conjugated ring with an intramolecular hydrogen bond in a quinoid structure is formed, *δ*_ΟΗ_ is deshielded, *i.e.*, the hydrogen bond is stronger than that in comparable aromatic systems. Thus, compounds **8** and **10** differ little in their *δ*_ΟΗ _values, whereas *δ*_ΟΗ_ in quinoid structures is considerably shielded. On the other hand, a conjugated six-membered aromatic system ring which does not contain the C=O group has a weaker intramolecular hydrogen bond (see **9** in Table 1). These changes in intramolecular hydrogen bond strengths can be explained in terms of the effect exerted on the π-electron distribution by formation of an intramolecular hydrogen bond to give a six-membered conjugated ring, which has been shown to play a very significant role [26]. *δ*_ΟΗ_ values in the six-membered rings of compounds **14** and **15** demonstrate a considerable intramolecular hydrogen bond strengthening in comparison with the other compounds in Table 1, presumably, due to the large effect of bond angles and lengths [11]. 

**Scheme 1:**
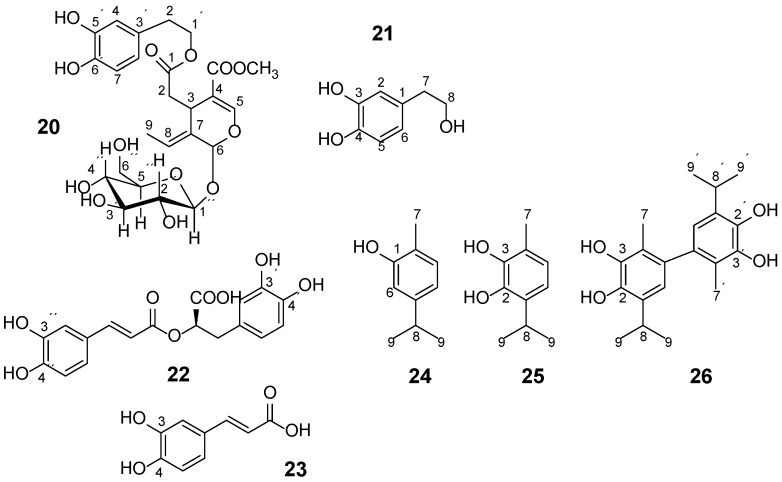
Chemical structure of oleuropein, **20**, hydroxytyrosol, **21**, rosmarinic acid, **22**, caffeic acid, **23**, carvacrol, **24**, *p*-cymene-2,3-diol, **25**, and *p*-cymene-2,3-diol 6-6′ dimer, **26** [25].

Kontogianni *et al.* [25] investigated in detail correlations between hydrogen bonds and solvent effects of phenol –OH chemical shifts for numerous phenolic acids, flavonoids and oleuropein derivatives (Table 2 and Scheme 1). The C-5 OH resonance of flavonoids is the more deshielded, it is practically solvent and temperature independent and appears in the region of 11.8 to13.5 ppm. The planar configuration of the six-membered ring and the π-delocalization of the hydrogen bonded heteroconjugated fragment result in a resonance-assisted [27,28] strong intramolecular hydrogen bond interaction, which results in a significant deshielding and reduction of the exchange rate of the OH proton. The C-5 OH resonance in DMSO-*d*_6_ is more deshielded in the presence of a C-2–C-3 double bond: 13.00 and 12.99 ppm for luteolin, **2**, and apigenin, **7**, respectively, compared to those molecules without a C-2–C-3 double bond e.g., 12.17 ppm and 12.18 ppm for eriodictyol, **13**, and naringenin, **12**, respectively (Table 2). This was attributed to the extensive conjugation of the C-2–C-3 double bond and the ring C with the OC-4 carbonyl group which results in a more polarizable CO bond and, thus, a stronger intramolecular hydrogen bond. The C-5 OH resonance is more deshielded in the flavones e.g., apigenin (13.00 ppm) and luteolin (13.01 ppm) compared to those of the flavonols quercetin (12.52 ppm) and kaempferol (12.45 ppm). This was attributed to the presence of the C-3 OH group of quercetin that attenuates the electron density of the OC-4 carbonyl oxygen atom and, thus, decreases the strength of the C-5 OH•••O C-4 intramolecular hydrogen bond. The same tendencies of the C-5 OH resonances have been observed for flavonoids in the absence of the C-2–C-3 double bond. The C-5 OH resonances of the flavones naringenin (12.20 ppm) and eriodictyol (12.19 ppm) are also more deshielded compared to those of aromadendrin, **14** (11.96 ppm) and taxifolin, **15** (11.96 ppm) [25].

**Table 1:** Chemical shifts of OH (*δ*_ΟΗ_) and CH (*δ*_CH_) groups in a series of quinoid compounds, in CDCl_3_ solutions [11].

No.	Compound	*δ*_ΟΗ_	*δ*_CH_	Conc., mol.%	No.	Compound	*δ*_ΟΗ_	Conc., mol.%
**1**	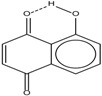	11.83	6.90	0.4	**9**	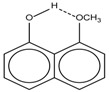	10.73	0.2
**2**	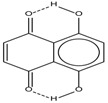	12.36	7.12	0.2	**10**	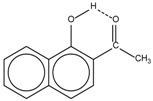	13.92 †	3.3 †
**3**	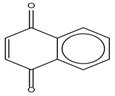	-	6.95	1.0	**11**	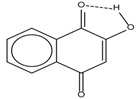	7.33	0.3
**4**	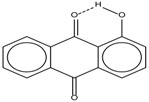	12.52	-	0.9	**12**	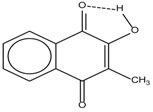	7.35	0.5
**5**	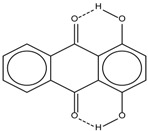	12.85	7.29	0.4	**13**	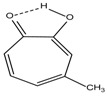	9.00	0.5
**6**	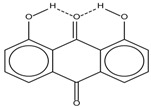	12.00	-	0.2	**14**	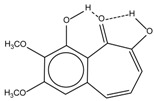	a 8.49 b 14.71	1.0
**7**	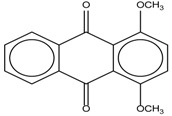	-	7.35	0.6	**15**	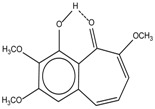	15.16	1.0
**8**	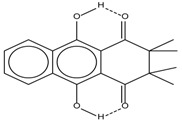	13.50	-	0.8				

† Dissolved in CCl_4_. a, b, stand for five-membered and six-membered conjugated rings with intramolecular hydrogen bonds, respectively.

**Table 2:** Structures of flavonoids [25]. 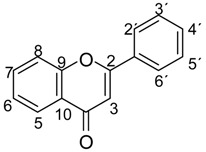

Compound	3	5		6		7			8		3′		4′		5′		C-2−C-3	
Quercetin (**1**)	OH	OH		H		OH			H		OH		OH		H		double	
Luteolin (**2**)	H	OH		H		OH			H		OH		OH		H		double	
Myricetin (**3**)	OH	OH		H		OH			H		OH		OH		OH		double	
Kaempferol (**4**)	OH	OH		H		OH			H		H		OH		H		double	
Chrysin (**5**)	H	OH		H		OH			H		H		H		H		double	
Genkwanin (**6**)	H	OH		H		OCH_3_			H		H		OH		H		double	
Apigenin (**7**)	H	OH		H		OH			H		H		OH		H		double	
4′,5,6-Trihydroxy-7-methoxyflavone (**8**)	H	OH		OH		OCH_3_			H		H		OH		H		single	
5,6-Dihydroxy-4′,7-dimethoxyflavone (**9**)	H	OH		OH		OCH_3_			H		H		OCH_3_		H		single	
5,6-Dihydroxy-3′,4′,7-trimethoxyflavone (**10**)	H	OH		OH		OCH_3_			H		OCH_3_		OCH_3_		H		single	
4′,5,6-Trihydroxy-7,8-dimethoxyflavone (**11**)	H	OH		OH		OCH_3_			OCH_3_		H		OH		H		single	
Naringenin (**12**)	H	OH		H		OH			H		H		OH		H		single	
Eriodictyol (**13**)	H	OH		H		OH			H		OH		OH		H		single	
Aromadendrin (**14**)	OH	OH		H		OH			H		H		OH		H		single	
Taxifolin (**15**)	OH	OH		H		OH			H		OH		OH		H		single	
Tamarixetin (**16**)	OH	OH		H		OH			H		OH		OCH_3_		H		double	
3′,4′,5,7-Tetrahydroxy-3-methoxyflavone (**17**)	OCH_3_	OH		H		OH			H		OH		OH		H		double	
3′,5,7-Trihydroxy-3,4′-dimethoxyflavone (**18**)	OCH_3_	OH		H		OH			H		H		OCH_3_		H		double	
3,5,7-Trihydroxy-3′,4′,5′-trimethoxyflavone (**19**)	OH	OH		H		OH			H		OCH_3_		OCH_3_		OCH_3_		double	

From an extensive search of the Cambridge Crystallographic Database (CSD) the dihedral angle ω_2_(Ο(5)–C(5)–C(10)–C(4)) in flavonoids [25] was found to vary from −4.12° to 2.87° and, thus, is essentially planar. The dihedral angle ω_1_(H–Ο(5)–C(5)–C(10)) shows a large variation from −30.13° to +13.12° which presumably indicates the difficulty in locating the position of exchangeable hydrogen atoms by the use of X-ray crystallography. The d_2_(C=O•••O(5)) contact varies from 2.582 Å to 2.697 Å and the d_1_(C-5 OH•••OC-4) contact from 1.678 Å to 2.179 Å which indicate the formation of a strong near planar intramolecular hydrogen bond. These results are in excellent agreement with the NMR data of the C-5 OH protons which are strongly deshielded.

The C-7 OH groups in DMSO-*d*_6_ appear in a very narrow and distinct region of 10.80 to 11.15 ppm. The presence of a double bond in position C-2–C-3 and the nature of substitution at the position 3 of the ring C appear to have a minor effect. The C-3 OH groups of flavonoids appear in two distinct regions. Compounds with a double bond in position C-2–C-3 (**1**, **3**, **4**, **16** and **19**) appear in the region of 9.3 to 9.7 ppm, while those without a C-2–C-3 double bond (**14** and **15**) appear at ~5.8 ppm which is the region of alcohol OH groups [25].

**Figure 8:**
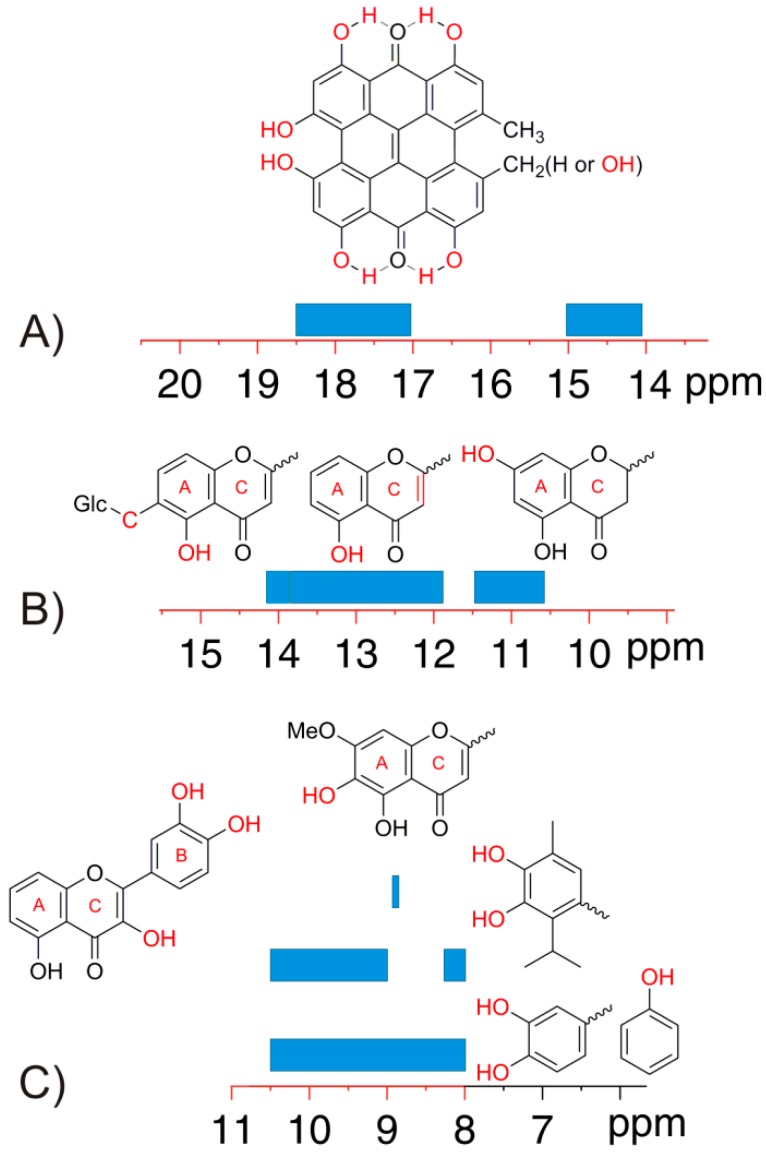
Classification of the chemical shifts of the –OH groups in DMSO-*d*_6_ on the basis of the structure of the molecule and the nature of the substituent in the rings. (**A**), (**B**) and (**C**) are the 15–20 ppm, 10–15 ppm, and 8–10 ppm regions, respectively.

The C-4′ OH group exhibits the most variable chemical shift range of 8.8 to 10.6 ppm; it is strongly shielded in myricetin, **3**, due to the presence of C-3′ OH and C-5′ OH groups, and it is strongly deshielded in the case of long range conjugation either to a C-2–C-3 double bond and a OC-4 carbonyl group (the case of apigenin) or a double bond due to substitution in the *para* position (the case of caffeic acid and rosmarinic acid). The C-4′ OH is furthermore deshielded by 0.6 to 1 ppm in the absence of OH groups in *ortho* positions such as in compounds **4**, **6**, **7**, **8** and **11**. In contrast, the chemical shift of the C-3′ OH groups appears in a narrow range of 9.05 to 9.50 ppm, and the presence of –OH groups in *ortho* positions has a minor effect. The C-6 OH groups of flavonoids with double bond C-2–C-3 and with R= –H in position 3 of ring C, CH_3_O–in position 7 and –H in position 8 of the ring A, respectively, appear at ~8.80 ppm. The –OH groups of simple phenols appear in the range of 8 to 9 ppm, e.g., **24**, as well as –OH groups located in *ortho* positions to each other in the phenolic ring, such as derivatives of carvacrol (**25** and **26**) and hydroxycinnamic acids (caffeic acid and rosmarinic acid) (Figure 8) [25].

**Figure 9:**
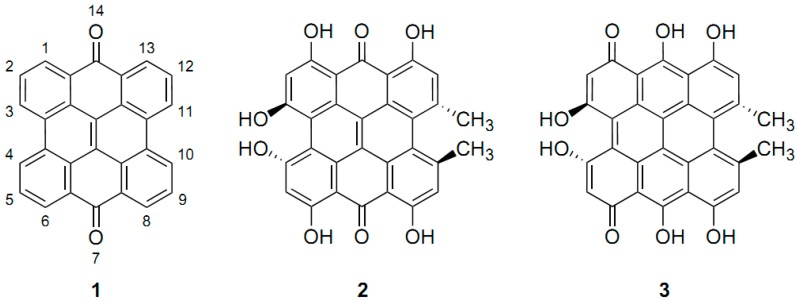
*meso*-Napthodianthrone skeleton-Q^7,14^ (**1**), 7,14-dioxo tautomer of hypericin (**2**), and 1,6-dioxo tautomer (**3**) [31].

**Figure 10:**
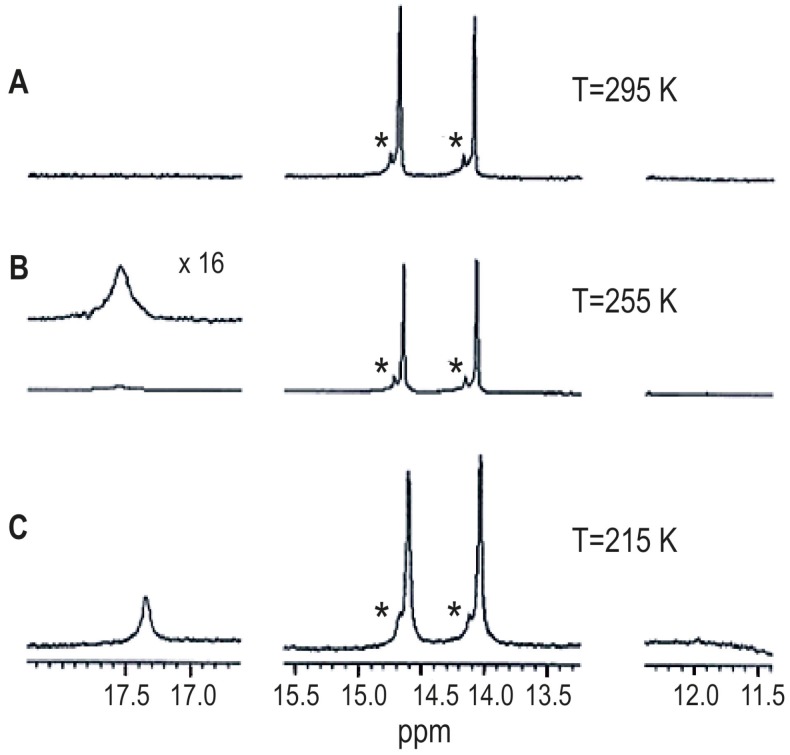
Variable temperature gradient ^1^H-NMR spectra of hypericin in MeOH-*d*_3_ (anionic form **4**): number of scans 64, 762 and 1312 for A, B and C, respectively. The asterisk denotes an unknown compound. Reprinted with permission from [31]. Copyright 2002, by Elsevier Science Ltd. (Amsterdam, The Netherlands).

**Figure 11:**
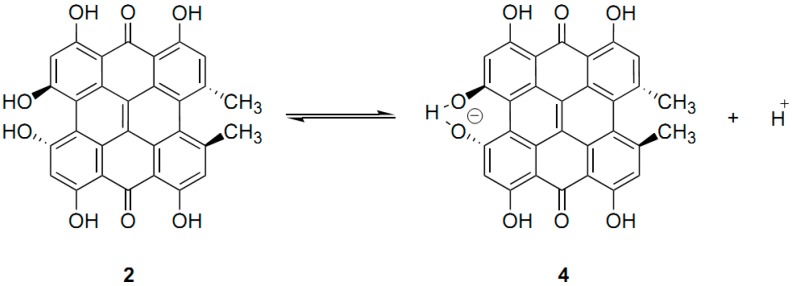
Solvent dependent equilibrium of 7,14 -dioxo tautomer of hypericin HyH, Q^7,14^ (**2**), and its anionic stable form Hy^−^ (**4**) [31].

Hypericin, a *meso*-napthodianthrone derivative (Figure 9), is a natural product that has drawn increased attention in the last decades due to its biochemical and photodynamic properties [29,30]. Hypericin has been assigned as the tautomer with the carbonyl groups in positions 7 and 14 (Q^7,14^ tautomer). The positions 3,4 and 10,11 are denoted as bay regions and those in positions 6,7,8 and 1,13,14 as *peri*-regions. The ^1^H-NMR spectrum of hypericin in acetone-*d*_6_ at room temperature displayed two hydroxyl signals (14.17 ppm, C-8 OH and C-13 OH) and 14.75 ppm (C-1 OH and C-6 OH) [31]. No signals for the bay hydrogens were recorded. However, at 215 K a broad line at *δ* = 11.6 to 11.8 ppm was detected and was assigned to the two bay hydroxyls. The ^1^H-NMR spectrum of hypericin at 295 K in MeOH-*d*_3_ (Figure 10) displayed two hydroxyl signals: 14.09 ppm (C-8 and C-13 OH) and 14.68 ppm (C-1 and C-6 OH). At low temperatures a broad peak at *δ* = 17.3 to 17.5 ppm, corresponding to the integral of one proton was recorded, and was assigned to the C-3/C4 OH of the hypericinate ionic form of **4** (Figure 11). This peak has also been recorded at 18.3 ppm in DMSO-*d*_6_ [32] contrary to earlier assignment [33] that a broad line at 8.2 ppm in DMSO-*d*_6_ should be attributed to the bay hydroxyl protons. Interestingly, hypericin crystallizes from pyridine in the form of an ion pair of the 3-hypericinate ion and the pyridinium cation [34]. The facile ionization of **2** (Figure 11) takes place at the OH in the bay region, thus, resulting in a very short distance of the O(3) and O(4) oxygens of 2.36 Å with one hydrogen atom bound to both of them. The deshielding of the C-3/C-4 OH of the hypericinate ionic form of **4** is in excellent agreement with the strong deshielding and, thus, very strong hydrogen bond of sodium 4,5-dihydroxynaphthalene-2,7-disulphonate at low temperature (*δ*(OH) = 17.72 ppm) [35] and of phenols which are involved in catalytic triads e.g. in ketosteroid isomerase (*δ*(OH) = 18.2 ppm) [36,37]. Similarly a strong hydrogen bond (δ = 18.4 ppm) has been observed in the heteroconjugated hydrogen bonded anion A•••H•••X^−^ of 2-chloro-4-nitrophenol with 3-phenylpropionic acid in CD_2_Cl_2_ at 175 K [21].

Interestingly, Bertolasi *et al.* [27] suggested a linear correlation between crystallographic data and *δ*(OH) ^1^H chemical shifts of the form:

*δ*(OH, ppm) = −34.1(±2.6) R(O•••O)(Å) + 100.3(±64.0) (1)

for a variety of molecules where the π-conjugated •••O=C–C=C–OH•••*β*-diketone enol group was found to form intramolecular O–H•••O hydrogen bonds. Correlation of R[(O)H•••O] hydrogen bond distances from X-ray diffraction with *δ*(^1^H) from solid state NMR of the form:

R[(O)H•••O] = 5.04 − 1.16ln*δ*(^1^H) + 0.0447*δ*(^1^H) (2)

was also suggested for crystalline aminoacids [38].

Variable temperature ^1^H-NMR studies were used to investigate a rare example of medium ring size intramolecular 9-membered hydrogen bonding between aromatic carbonyl and phenolic hydroxyl groups of 2-arylmethylphenols having carbonyl groups at 2′-position in CDCl_3_ solution [39]. Substituent effects of these compounds indicated that the strength of intramolecular hydrogen bonding depends on the basicity of the carbonyl group and the acidity of the phenol group. 

Castellano *et al.* [40] investigated in detail, through Cambridge Structural Database (CSD) and literature mining, the occurrence of 7- membered intramolecular hydrogen bonding involving phenol hydroxyl group donors. The CSD revealed numerous accepting functional groups including alcohols, ethers, amides, amines, ketones, N- containing heterocycles, N- oxides, phosphates, and phosphane oxides, with O (phenol)•••N and O (phenol)•••O distances of c.a. 2.7 Å and two dihedral angles within the range of typical *γ*- turns in peptides. The phenolic –OH resonances in CDCl_3_ were found to be strongly deshielded by 3.6 to 7.9 ppm with respect to the non- hydrogen bonded monomeric 2-isopropylphenol (*δ* = 4.7 ppm). 

### 4.2. Effects of Solvents

The chemical shifts of the –OH group of phenol in DMSO-*d*_6_ (9.36 ppm), acetone-*d*_6_ (8.29 ppm), CD_3_CN (6.90 ppm), and CDCl_3_ (4.65 ppm) clearly demonstrate that the magnitude of the shifts is related to the strength of hydrogen bonding of the solvent with a differential solvent effect Δ*δ* = *δ*(DMSO-*d*_6_) − *δ*(CDCl_3_) = 4.71 ppm [41]. In the case of 4-methylcatechol the two OH groups in an *ortho* relationship were found to be more shielded compared to the OH group of phenol. The differential solvent effects Δ*δ* = *δ*(DMSO-*d*_6_) − *δ*(acetone-*d*_6_) of 0.99 and 1.06 ppm and Δ*δ* = δ(DMSO-*d*_6_) − *δ*(CD_3_CN) of 2.15 and 2.19 ppm for the C-1 OH and C-2 OH groups of 4-methylcatechol were found to be similar to that of phenol. This demonstrates no significant solvation differences between the C-1 OH and C-2 OH groups of 4-methylcatechol compared to that of phenol. In contrast, significantly smaller chemical shift differences were observed for C-1 OH and C-2 OH groups of 4-methylcatechol between DMSO-*d*_6_ and CDCl_3_ (Δ*δ* = 3.69 ppm), compared to that in phenol (Δ*δ* = 4.71 ppm) which should be attributed to an intramolecular flip-flop hydrogen bonding [1,42] in CDCl_3_ solution. 

Table 3 illustrates the chemical shifts of hydroxyl protons of quercetin (**1**) kaempferol (**4**) and genkwanin (**6**) in DMSO-*d*_6_, acetone-*d*_6_ and CD_3_CN [25]. The shielding differences between DMSO-*d*_6_ and CD_3_CN are in the range of 2.30 to 2.86 ppm for the C-7, C-3, C-3′, and C-4′ OH of quercetin, C-7, C-3, and C-4′ OH of kaempferol and C-4′ of genkwanin. In contrast, very small shielding differences Δ*δ*[(DMSO-*d*_6_)−(CD_3_CN)] ≤0.6 ppm were observed for the C-5 OH protons. Due to intramolecular C-5 OH•••OC-4 hydrogen bonding the surrounding solvent molecules around the C-5 hydroxyl proton are excluded, leading to a significantly reduced solvation and a consequent small shielding solvent dependence relative to that of C-7, C-3′, and C-4′ OH protons. The presence, therefore, of intramolecular hydrogen bond interactions can be deduced from the solvent dependent chemical shifts of hydroxyl proton signals. 

**Table 3:** Chemical shifts of hydroxyl protons of quercetin (**1**), kaempferol (**4**), genkwanin (**6**), oleuropein (**20**), and hydroxytyrosol (**21**) (Table 2) in DMSO-*d*_6_, acetone-*d*_6_ and CD_3_CN and their differences at 298 K [25].

Compound		*δ*_DMSO__-_*_d_*_6_	*δ*_Acetone__-_*_d_*_6_	Δ*δ*(*δ*_DMSO__-_*_d_*_6_ − *δ*_Acetone__-_*_d_*_6_)	*δ*_CD3CN_	Δ*δ*(*δ*_DMSO__-_*_d_*_6_ − *δ*_CD3CN_)
Quercetin (**1**)	C-5 OH	12.52	12.18	0.34	12.01	0.51
	C-7 OH	10.80	9.61	1.19	8.11	2.69
	C-3 OH	9.37	7.97	1.40	7.02	2.35
	C-3′ OH	9.32	8.41	0.91	7.02	2.30
	C-4′ OH	9.61	8.58	1.03	7.27	2.34
Kaempferol (**4**)	C-5 OH	12.45	12.19	0.26	12.02	0.43
	C-7 OH	10.82	9.63	1.19	8.00	2.82
	C-3 OH	9.37	8.03	1.34	7.01	2.36
	C-4′ OH	10.11	9.00	1.11	7.59	2.52
Genkwanin (**6**)	C-5 OH	12.95	13.03	−0.08	12.95	0.00
	C-4′ OH	10.56	9.26	1.30	7.70	2.86
Oleuropein (**20**)	C-5′ OH	8.74	7.75	0.99	6.71	2.03
	C-6′ OH	8.68	7.73	0.95	6.56	2.12
	C-2′′ OH	5.14	4.46	0.68	3.48	1.66
	C-3′′ OH	5.02	4.32	0.70	3.48	1.54
	C-4′′ OH	4.96	4.25	0.71	3.37	1.59
	C-6′′ OH	4.48	3.67	0.81	2.92	1.56
Hydroxytyrosol (**21**)	C-3 OH	8.72	7.68	1.04	6.53	2.19
	C-4 OH	8.63	7.65	0.98	6.51	2.12
	C-8 OH	4.60	3.54	1.06	2.60	2.00

From an extensive search of the Cambridge Crystallographic Database (CSD) [25] an intramolecular hydrogen bond was suggested in flavonoids between C-3 OH and OC-4, resulting in the formation of a five-membered ring. The dihedral angle ω_4_(Ο(3)–C(3)–C(4)–O(4)) varies from −1.92° to +3.94° and, thus, is essentially planar. The dihedral angle ω_3_(H–Ο(3)–C(3)–C(4)) shows a larger variation from −9.46° to +18.66° which may be attributed to the uncertainty in locating labile protons in the X-ray structures. The d_4_(C=O•••O(3)) contact varies from 2.686 Å to 2.764 Å, which is on average longer than the d_2_(C=O•••O(5)) contact, and the d_3_(C=O•••H–O(3)) contact from 2.169 Å to 2.452 Å. Domagala *et al.* [43] very recently provided a charge-density analysis of accurate high-resolution single-crystal X-ray diffraction data of quercetin monohydrate. The O3–H3 hydroxyl group is the most out-of-plane of the aromatic ring with dihedral angles H3–O3–C3–C4 of −14.62°, −14.45° and −11.26° compared with the H5–O5–C3–C4 dihedral angles of −0.53°, 2.1° and 4.71°. Furthermore, the TAAM_OPT deformation electron density map appeared to be slightly smeared which might imply a partial sp^3^ hybridization of the O3 atom.

Contrary to the X-ray results, the C-3 OH protons of quercetin (**1**) and kaempferol (**4**) exhibit the largest shielding difference between DMSO-*d*_6_ and acetone-*d*_6_ solution among the –OH groups of the compounds in Table 3. This demonstrates that the C-3 OH is solvent accessible and does not form a persistent intramolecular hydrogen bond with the OC-4 of appreciable strength. The possibility that the above intramolecular hydrogen bond exists transiently cannot be excluded especially in CD_3_CN solution [25].

### 4.3. Temperature Effects of Phenol OH Protons

**Figure 12:**
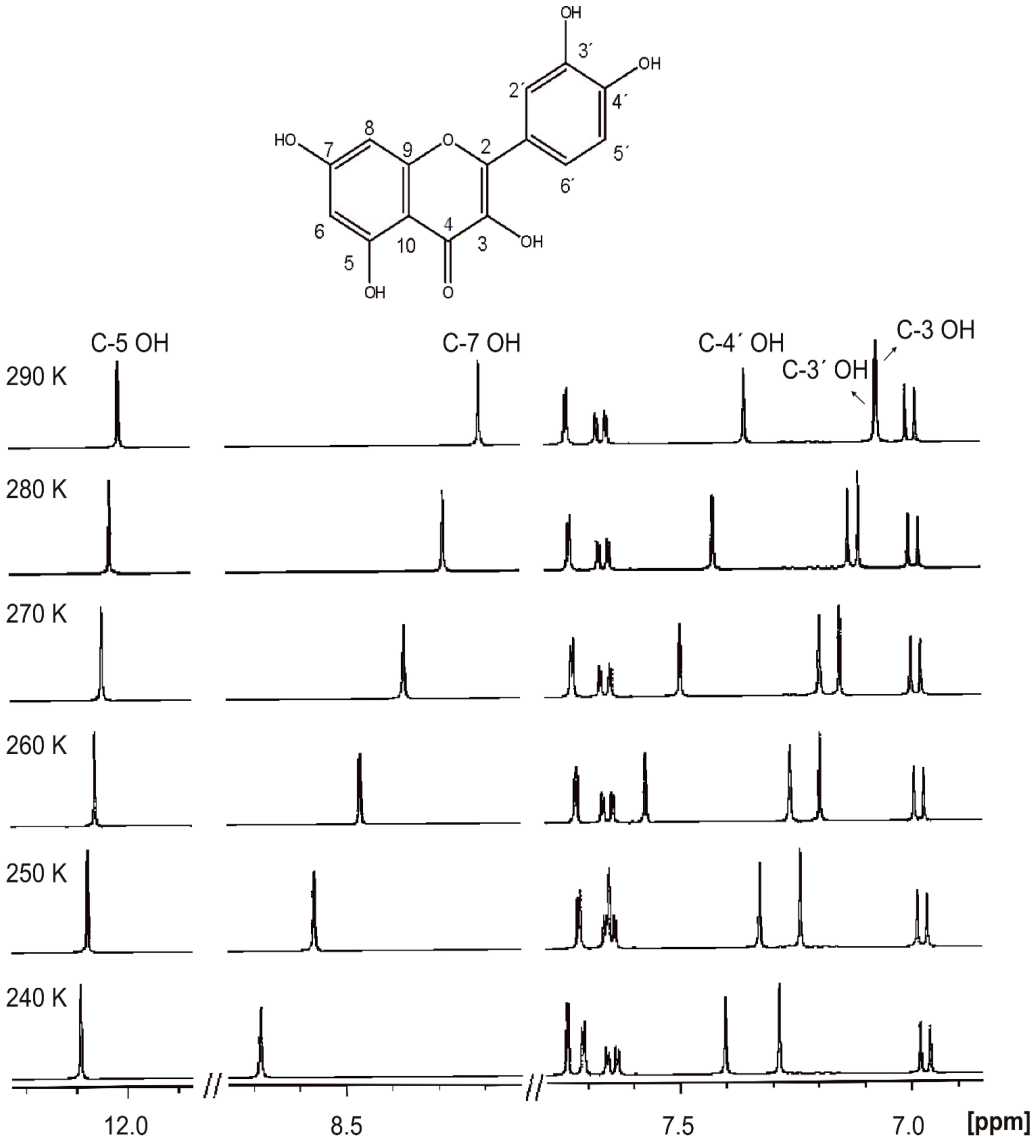
Variable temperature (400 MHz) ^1^H-NMR spectra of quercetin in CD_3_CN, concentration 5 mM, with the presence of 2 μL picric acid, 8 mM in CD_3_CN (number of scans = 32, experimental time = 4 min). Reprinted with permission from [25]. Copyright 2013, The Royal Society of Chemistry.

Figure 12 shows the temperature dependence of the hydroxyl protons chemical shifts, Δ*δ*/ΔT, of quercetin in CD_3_CN. In general the OH resonances are shifted to low frequency as the temperature increases (negative temperature coefficients). Table 4 lists Δ*δ*/ΔT values of the –OH protons of quercetin, kaempferol, genkwanin, oleuropein and hydroxytyrosol in DMSO-*d*_6_, acetone-*d*_6_, and CD_3_CN. The temperature dependent changes in chemical shifts are linear and the derived Δ*δ*/ΔT coefficients, with the coefficient of linear regression R^2^ > 0.995, span a range of −0.5 to −12.3 ppb K^−1^ [25]. Δ*δ*/ΔT values in DMSO-*d*_6_, with the exception of C-5 OH of flavonoids, are in the range of −5.4 to −8.0 ppb K^−1^ when reference is made to the solvent peak (Table 4). This range of Δ*δ*/ΔT values implies that all the phenol –OH groups are exposed to the solvent and the –OH groups in the *ortho* position, such as the C-3′ and C-4′ OH groups of quercetin, are not involved in intramolecular flip-flop hydrogen bond interactions [25,42]. 

Δ*δ*/ΔT values in acetone-*d*_6_, with the exception of C-5 OH flavonoids, are in the range of −8.4 to −11.8 ppb K^−1^ and, thus, larger in absolute terms than those in DMSO-*d*_6_. On increasing the temperature, the inter-molecular hydrogen bond with solute molecules is weakened and the hydroxyl proton is shielded. This implies that intermolecular solute-solvent hydrogen bonding interactions in acetone-*d*_6_ are broken more easily than in DMSO-*d*_6_ by increasing the temperature. Δ*δ*/ΔT values in CD_3_CN are similar to those in DMSO-*d*_6_ with the exception of C-3 OH of quercetin (**1**) and kaempferol (**4**), which demonstrates the solvent accessibility of this group. Δ*δ*/ΔT values of C-5 OH (more positive than −2.5 ppb K^−1^) are significantly smaller in absolute terms than those of other hydroxyl protons which are exposed to the solvent, irrespective of the solvent used [25]. 

### 4.4. Theoretical Calculations

**Figure 13:**
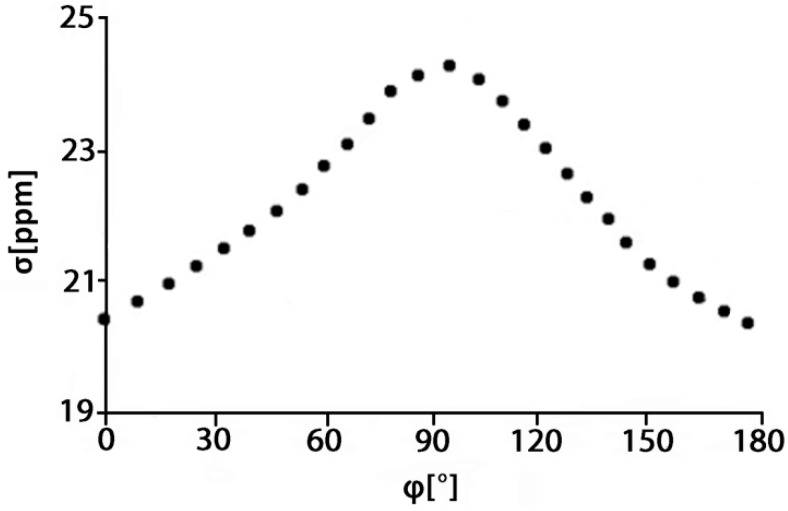
R–X═O•••H dihedral angle (ϕ) dependence of the nuclear shielding of the OH hydrogen of phenol. The hydrogen bond acceptor is acetone. Adapted with permission from [44]. Copyright 2007, by John Wiley & Sons, Ltd.

Abraham and Mobil [44] performed *ab initio* calculations of a model acetone-phenol system. The OH shielding was found to be linear with the H---O=C distance, R, for R < 2.1 Å with a shielding coefficient of −7.8 ppm Å^−1^ and proportional to cos^2^φ where φ is the H---O=C–C dihedral angle (Figure 13).

**Table 4:** Chemical shift temperature coefficients (Δ*δ*/ΔT)*^a,b^* of hydroxyl protons of quercetin (**1**), kaempferol (**4**), genkwanin (**6**), oleuropein (**20**), and hydroxytyrosol (**21**), in DMSO-*d*_6_, acetone-*d*_6_, and CD_3_CN solution. Reprinted with permission from [25]. Copyright 2013, The Royal Society of Chemistry.

Compound	Δ*δ*/ΔΤ *^a,b^*			
		DMSO-*d*_6_	Acetone-*d*_6_	CD_3_CN
Quercetin (**1**)	C-5 OH	−2.0 (−2.4)	−2.9 (−3.4)	−1.5 (−2.1)
	C-7 OH	−5.4 (−5.8)	−11.1 (−11.6)	−9.9 (−10.5)
	C-3 OH	−8.0 (−8.4	−11.6 (−12.1)	−4.4 (−5.0)
	C-3′ OH	−6.3 (−6.7)	−8.4 (−8.9)	−6.7 (−7.3)
	C-4′ OH	−6.5 (−6.9)	−11.2 (−11.7)	−8.0 (−8.6)
Kaempferol (**4**)	C-5 OH	−2.0 (−2.4)	−3.1 (−3.6)	−1.3 (−1.9)
	C-7 OH	−5.8 (−6.2)	−10.7 (−11.2)	−9.6 (−10.2)
	C-3 OH	−8.2 (−8.6)	−11.8 (−12.3)	−4.1 (−4.7)
	C-4′ OH	−5.7 (−6.1)	−10.1 (−10.6)	−6.7 (−7.3)
Genkwanin (**6**)	C-5 OH	−2.1 (−2.5)	−1.4 (−1.8)	−0.8 (−1.4)
	C-4′ OH	−8.9 (−9.3)	−8.9 (−9.3)	−8.5 (−9.1)
Oleuropein (**20**)	C-5′ OH	−7.0 (−7.4)	−9.2 (−9.7)	−7.5 (−8.1)
	C-6′ OH	−6.9 (−7.3)	−9.1 (−9.6)	−7.3 (−7.9)
	C-2′′ OH	−6.9 (−7.3)	−9.6 (−10.1)	−5.6 (−6.2)
	C-3′′ OH	−7.0 (−7.4)	−8.7 (−9.2)	−5.0 (−5.6)
	C-4′′ OH	−6.2 (−6.6)	−8.2 (−8.7)	−5.0 (−5.6)
	C-6′′ OH	−6.3 (−6.7)	−8.7 (−9.2)	−7.2 (−7.8)
Hydroxytyrosol (**21**)	C-3 OH	−7.8 (−8.2)	−9.6 (−10.1)	−6.7 (−7.3)
	C-4 OH	−7.6 (−8.0)	−9.3 (−9.8)	−6.3 (−6.9)
	C-8 OH	−6.2 (−6.6)	−8.7 (−9.2)	−5.3 (−5.9)

*^a^* Expressed in parts per 10^9^ (ppb) per K (relative to the solvent resonance). *^b^* The values into parenthesis were corrected taking into consideration the value of Δ*δ*/ΔΤ of the solvent having as a reference internal standard TMS for acetone-*d*_6_ and CD_3_CN, and TMSP-*d*_4_ for DMSO-*d*_6_.

Siskos *et al.* [41] performed extensive calculations with the polarizable continuum model (PCM) [45] and PCM discrete phenol + solvent hydrogen bonded complexes. The calculated phenolic OH proton chemical shifts with the polarized continuum model (*δ*_ΟH_(CHCl_3_) = 4.27 ppm, *δ*_ΟH_(MeCN) = 4.44 ppm, *δ*_ΟH_(acetone) = 4.42 ppm and *δ*_OH_(DMSO) = 4.44 ppm) were found to deviate significantly from the experimental values (*δ*_ΟH_(CDCl_3_) = 4.65 ppm, *δ*_ΟH_(CD_3_CN) = 6.90 ppm, *δ*_ΟH_(acetone-*d*_6_) = 8.29 ppm, and *δ*_OH_(DMSO-*d*_6_) = 9.36 ppm, especially in the case of solvents of high dielectric constant and hydrogen bonding strength.

This may be attributed to the fact that this model is the quantum mechanical formulation of the Onsager reaction field model and does not include specific solvent–solute interactions like hydrogen bonds. In contrast, the DFT method with the B3LYP hybrid functional [46] 6-31G(d), 6-31+G(d) and 6-311++G(d,p) basis set, as implemented in the Gaussian 03 package, resulted in an excellent improvement of the calculated OH chemical shift when using discrete PhOH + solvent complexes. The GIAO method with the DFT/B3LYP/6-311++G(2d,p) functional was implemented by minimizing a discrete complex between PhOH and a single solvent molecule in the gas phase using either the DFT/B3LYP/6-31+G(d) or DFT/B3LYP/6-311++G(d,p) basis set (Table 5).

**Table 5:** Calculated OH ^1^H-NMR chemical shifts (relative to TMS, ppm) of 1:1 PhOH + solvent complexes with the gauge invariant atomic orbitals (GIAO) method at the DFT/B3LYP/6-311++G(2d,p) level of theory in the gas phase and by using the CPCM model [41].

1:1 PhOH+ solvent complex	DFT/B3LYP 6-31+G(d) geometry optimization, gas phase	DFT/B3LYP 6-31+G(d) geometry optimization, CPCM	DFT/B3LYP 6-311++G(d,p) geometry optimization, gas phase	DFT/B3LYP 6-311++G(d,p) geometry optimization, CPCM	Experimental values
PhOH + CHCl_3_	3.96	4.61	3.85	4.49	4.65
PhOH + MeCN	6.43	6.80	6.42	6.79	6.90
PhOH + acetone	8.60	8.95	8.48	8.83 8.71 *^a^*	8.29
PhOH + DMSO	9.02	9.31	9.08	9.37	9.36

^a^ Calculations using the DFT/B3LYP/ cc-pVTZ method.

**Figure 14:**
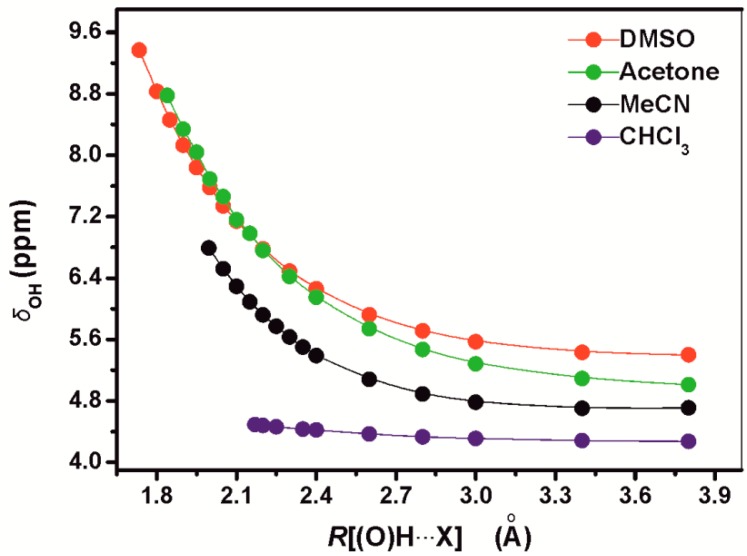
The dependence of the OH proton chemical shifts, *δ*OH (ppm), of the 1:1 phenol + solvent complexes *vs.* the distance R[(O)H•••X] (X = O for DMSO (red) and acetone (green), X = N for MeCN (black), and X = C for CHCl_3_(blue). Reprinted with permission from [41]. Copyright 2013, The Royal Society of Chemistry.

**Figure 15:**
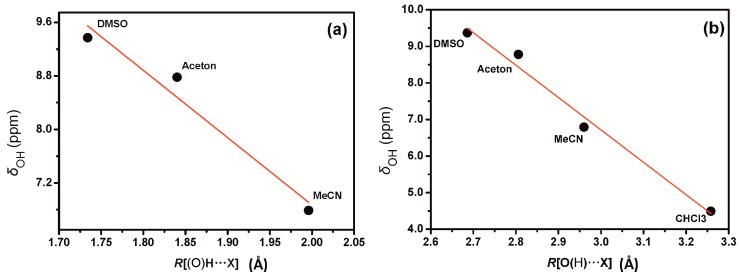
The dependence of the OH proton chemical shift, *δ*OH (ppm), in the minimum energy conformer, optimized at the DFT/B3LYP/6-311++G(d,p) level of theory, of 1:1 phenol + solvent complexes *vs.* R[(O)H•••X] (**a**) and R[O(H)•••X] (**b**) distances (X = O for DMSO and acetone, X = N for MeCN, and X = C for CHCl_3_). Reprinted with permission from [41]. Copyright 2013, The Royal Society of Chemistry.

Increasing the distance of the (O)H•••X hydrogen bond without changing any other parameter has a very significant effect in ^1^H chemical shifts with the exception of the 1:1 PhOH + CHCl_3_ complex (Figure 14). Beyond 2.5 Å the increase in nuclear shielding was found to be comparably moderate. For distances ≤2.1 Å the plots can be reproduced by a linear equation. The correlation coefficients (R^2^) for the 1:1 PhOH + DMSO and PhOH + acetone complexes were found to be 0.982 and 0.993 and demonstrate a very significant change in chemical shift of −6.08 ppm Å^−1^ and −6.22 ppm Å^−1^, respectively, upon increase in the (O)H•••X hydrogen bond length. In the minimum energy conformers of the PhOH+DMSO, PhOH+acetone, and PhOH+CH_3_CN complexes, the OH chemical shifts were found to exhibit a strong linear dependence on the R[(O)H•••X] hydrogen bond distance of −10.06 ppm Å^−1^ (R^2^ = 0.926) (Figure 15a). Similarly, linear dependence was observed for the phenol + solvent complexes (including the phenol + CHCl_3_ complex) on the R[O(H)•••X] hydrogen bond distances of −8.9 ppm Å^−1^ (R^2^ = 0.977) (Figure 15b). The linear correlation between crystallographic data and *δ*(OH) ^1^H chemical shifts suggested by Bertolasi *et al.* [27], therefore, may have a solid theoretical basis.

A very strong intramolecular hydrogen bond between the C-5 OH•••OC-4 groups was observed in all conformers. An attempt to investigate the interaction of a solvent molecule, like DMSO, with the C-5 OH group by the use of DFT calculations was unsuccessful. The DMSO molecule was displaced to a distance greater than 4 Å [41]. This is in excellent agreement with the experimental data which demonstrated very small chemical shift difference Δ*δ*[(DMSO-*d*_6_) − (CDCl_3_)] ≈ 0.02 ppm for the C-5 OH proton of genkwanin.

**Figure 16:**
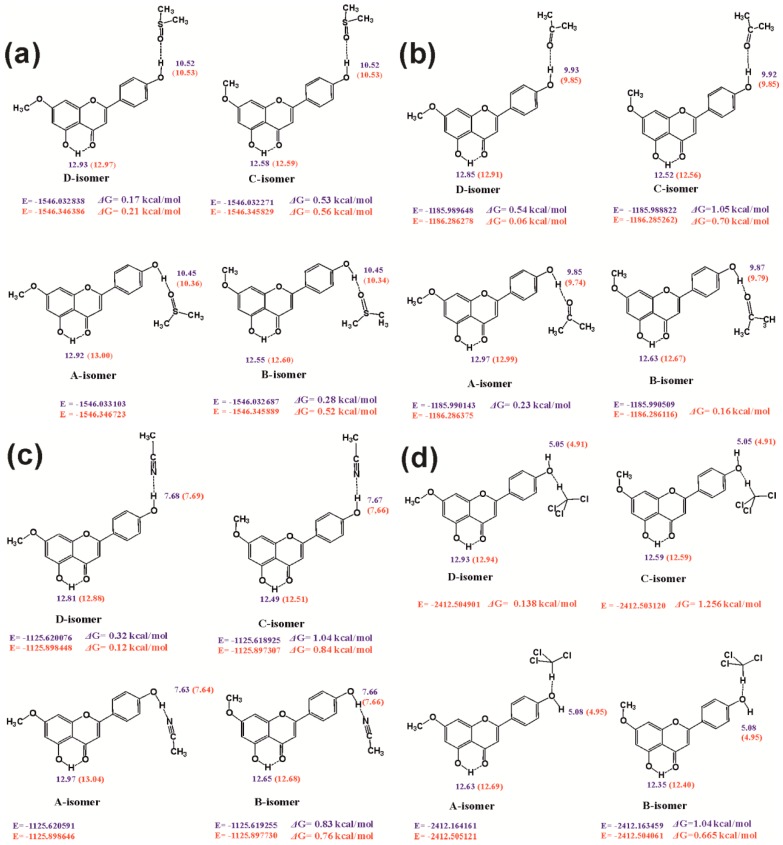
The effect of conformation of the C-7 OCH_3_ and C-4′ OH groups on the chemical shifts of the C-4′OH and C-5 OH protons for the 1:1 complexes of (**a**) genkwanin + DMSO; (**b**) genkwanin + Me_2_CO; (**c**) genkwanin + MeCN and (**d**) genkwanin + CHCl_3_, with the use of DFT/B3LYP/6-31+G(**d**) (data in blue) and DFT/B3LYP/6-311++G(d,p) (data in red) level of theory. Reprinted with permission from [41]. Copyright 2013, The Royal Society of Chemistry.

Figure 16 illustrates the effect of the conformation of the C-7 OCH_3_ and C-4′ ΟH groups on the chemical shifts of the C-4′ ΟH and C-5 OH protons, for the 1:1 complexes of genkwanin with DMSO, acetone, CH_3_CN, and CHCl_3_. 

The conformation of the C-4′ ΟH group has a minor effect (<0.08 ppm) on the chemical shift of the C-4′ ΟH proton for all the complexes studied. Similarly the conformation of the C-7 OCH_3_ group has a minor effect (≤0.03 ppm) on the C-4′ ΟH chemical shift. The conformation of the C-4′ ΟH group has a minor effect on the C-5 OH chemical shift in the DMSO complex (≤0.03 ppm) and a shielding effect ≤0.17 ppm in CH_3_CN and acetone complexes. In contrast, a significant effect of the conformation of the C-7 OCH_3_ group on the C-5 OH chemical shifts of 0.32 to 0.37 ppm was observed for all the solvation complexes. 

### 4.5. Calculated vs Experimental ^1^H Chemical Shifts

**Figure 17:**
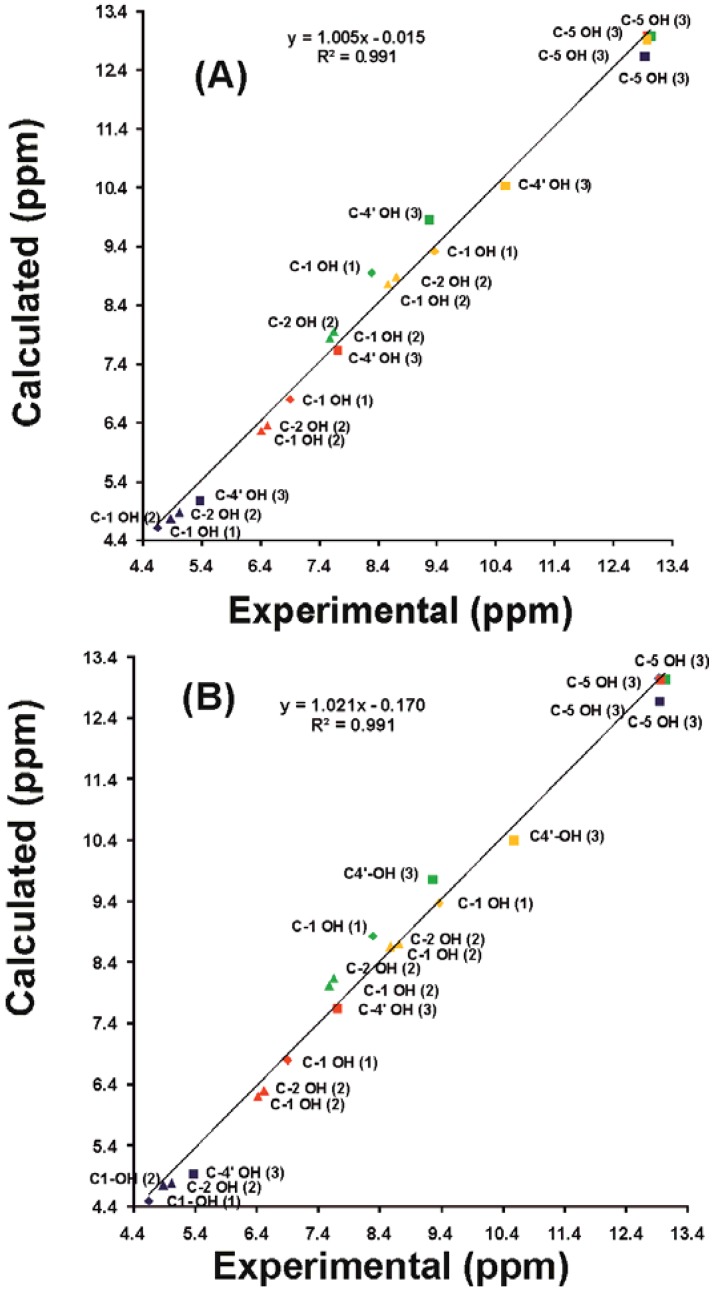
Calculated (at the GIAO DFT/B3LYP/6-311++G(2d,p) level of theory) *vs.* experimental values of the chemical shifts of the –OH protons of phenol, 4-methylcatehol and genkwanin in different solvents with minimization of the solvation complexes at the DFT/B3LYP/6-31+G(d) (**A**) and DFT/B3LYP/6-311++G(d,p) (**B**) level of theory. Reprinted with permission from [41]. Copyright 2013, The Royal Society of Chemistry.

**Figure 18:**
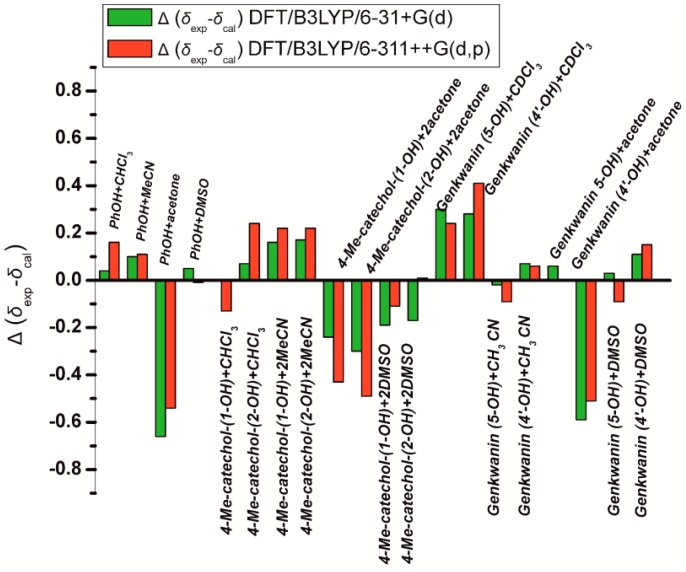
Histogram of the errors Δ(*δ*_exp_ − *δ*_cal_) of the calculated (at the GIAO DFT/B3LYP/6-311++ G(2d,p) level of theory) ^1^H OH chemical shifts with minimization of the solvation complexes at the DFT/B3LYP/6-31+G(d) and DFT/B3LYP/6-311++G(d,p) level of theory. Reprinted with permission from [41]. Copyright 2013, The Royal Society of Chemistry.

Figure 17 shows the parity plot and Figure 18 the histogram of the errors Δ(*δ*_exp_ − *δ*_cal_) between the experimental and the calculated (with the GIAO method at the DFT/B3LYP/6-311++G(2d,p) level) ^1^H solvent dependent chemical shifts with minimization of the solvation complexes at the DFT/B3LYP/6-31+G(d) and DFT/B3LYP/6-311++G(d,p) level of theory. The R^2^ value of 0.991 in both cases shows excellent correlation between the calculated solvent dependent isotropic chemical shifts of OH protons and the experimental chemical shifts. It may also be concluded that very large basis sets for energy minimization are not necessary to reproduce accurately ^1^H-NMR chemical shifts. The histogram in Figure 18 shows that the calculated ^1^H chemical shifts in acetone had errors larger than 0.3 ppm with the exception of the C-5 OH of genkwanin (the use of the DFT/B3LYP/6-311++G(d,p) level of theory was not able to reduce the error). The Δ(*δ*_exp_ − *δ*_cal_) error should be attributed to the strong tendency of acetone, which has no parallel with DMSO and CH_3_CN, to form C1–O–H•••O(C) dihedral angle which is strongly dependent on the basis set used [41].

Interestingly, the conformations with the minimum electronic energy and ΔG values of genkwanin in DMSO, acetone, CH_3_CN, and CHCl_3_ (Figure 16) show the best agreement between experimental and calculated chemical shift data of the C-5 OH proton. Therefore, the C-5 OH chemical shifts can be used as an internal sensor of the conformation state of substituents of ring A in the family of flavonoids and, presumably, to other classes of natural products.

## 5. Spin-Coupling Constants

**Figure 19:**
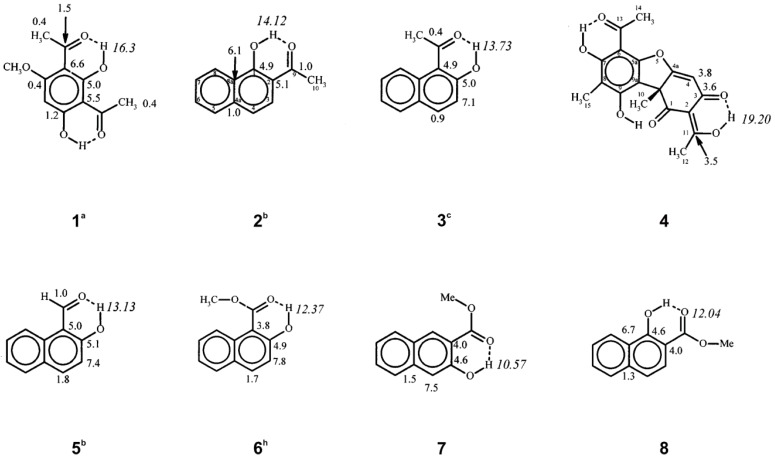
Observed OH chemical shifts (ppm) and *^n^J*(^13^C, O^1^H) coupling constants (Hz). ^a^ 300 K. ^b^ 265 K. ^c^ 250 K. ^h^ 290 K [50].

**Figure 20:**
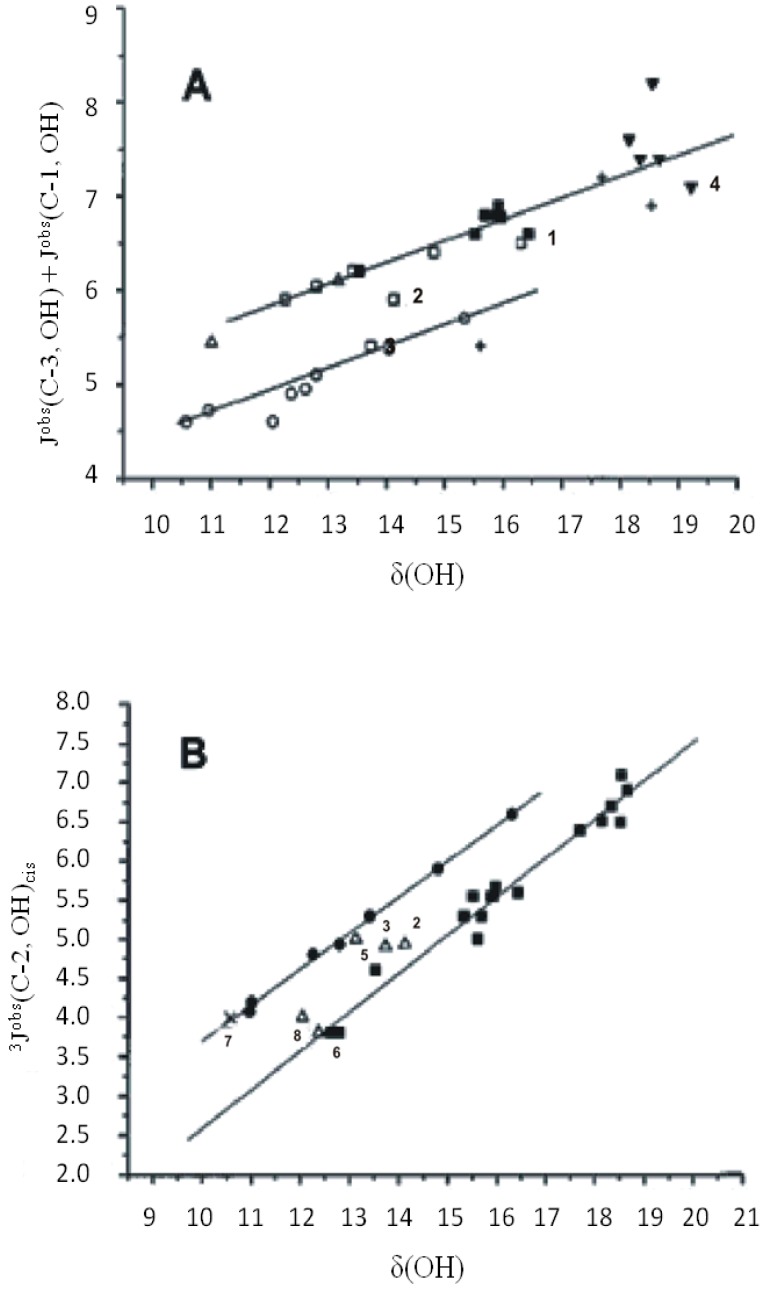
Plot of the *J*^obs^(C-3, OH) + *J*^obs^(C-1, OH) *vs* *δ*(OH). (**A**) □, ketones; ○, esters; ■, diketones; ▼, triketones; ∆, aldehydes; +, amides. (**B**) ■, Olefinic; ●, o-hydroxyacylbenzes; ∆, 1,2-disubstituted napthlalenes; X, 2,3-disubstituted napthlalenes. The numbers refer to the compounds of Figure 19. Adapted with permission [50]. Copyright 1998, John Willey & Sons.

Spin- coupling constants of phenol –OH protons (Figure 19) can be transmitted via the aromatic carbon skeleton or via hydrogen bonds, particularly in cases with long O – H and short O•••O distances leading to substantial orbital overlap. *^n^J*(C, OH) couplings have been investigated as tools for assignment, conformational analysis, determination of hydrogen bond strength, and to estimate the amount of each tautomer in tautomeric equilibria [47,48,49,50]. A plot of *J^obs^*(C-3, OH) + *J^obs^*(C-1, OH) *vs.* *δ*ΟΗ showed reasonable correlation both for compounds with localized hydrogen bonds and those displaying tautomerism (Figure 20). Furthermore, it was suggested that *J^obs^*(C-1', OH) and *J^obs^*(C-1'', OH) may be used for estimating the mole fractions of tautomeric systems [50].

## 6. Correlation of Phenol OH ^1^H Chemical Shifts with Heteronuclear Chemical Shifts

Jaccard and Lauterwein [51] investigated intramolecular hydrogen bonds of the type C=O ••• H–O in 1,4-napthoquinone, 5-hydroxy-1,4-naphtoquinone (juglone) and in a series of aromatic aldehydes and ketones. The ^17^O and ^1^H chemical shifts at the OH group show only a moderately good linear correlation (6 points, correlation coefficient R^2^ = 0.941). It should be emphasized that solvent effects on ^17^O chemical shifts of phenol compounds are surprisingly smaller than those of the carbonyl and amide oxygens and do not appear to correlate with hydrogen bonding strength of the solvents [52,53]. The ^13^C chemical shifts of the carbonyl groups do not show any functional relationship with the –OH ^1^H and ^17^O chemical shifts. 

Koeppe *et al.* [23] found a monotonous high frequency shift of the phenolic carbon in position 1 of heteroconjugated anions AHX^−^ of 1-^13^C-2-chloro-4-nitrophenol when the phenolic O–H distance is increased. In order to eliminate structural influences and highlight the effect of hydrogen bonding, the chemical shift of C-1 of the heteroconjugated anion was re-referenced with respect to the corresponding shift of the homoconjugated anion, usually also present in the solutions studied, as follows:

δ*(A H X) = *δ*(A H X) − *δ*(A H A) (3)

Plot of ^1^H chemical shifts of *δ*(A H X) of heteroconjugated complexes AHX^−^ of 1-^13^C-4-nitrophenol and 1-^13^C-2-chloro-4-nitrophenol in CD_2_Cl_2_ (175 K) and CDF_3_/CDF_2_Cl (120 K) as a function of δ*(A H X) demonstrated a bell-shaped correlation for more than 50 experimental data points. It was suggested that ^13^C chemical shifts can be a criterion for the phenolic oxygen-hydrogen distances. 

## 7. Phenol –OH Diffusion Coefficients

Diffusion-ordered spectroscopy (DOSY) is a sensitive NMR technique that allows the determination of self-diffusion coefficients [54] and, thus, can in principle be used to investigate the effect of intramolecular hydrogen bonding on the phenol –OH diffusion coefficients and lifetimes of hydroxyl protons. The DOSY spectra of a mixture of six flavonoids demonstrated that the C-7, C-3, C-3′ and C-4′ OH protons, which appeared as broad signals, have diffusion coefficients which are clearly different from those of the C-5 OH although they belong to the same molecule [55]. These protons, therefore, with apparent diffusion coefficients of 6.79 to 6.33 × 10^−1^^0^ m^2^ s^−1^, are similar to those of the residual H_2_O in the organic solvent (D_H_2_O_ = 6.81 × 10^−1^^0^ m^2^ s^−1^) indicating that they are in fast exchange with water. In contrast, the diffusion coefficients of the C-5 OH protons (1.89 to 2.17 × 10^−1^^0^ m^2^ s^−1^) are significantly smaller than those of the C-7, C-3, C-3′ and C-4′ OH protons. This should be attributed to the fact that the C-5 OH protons participate in a strong intramolecular C-5 OH•••OC-4 hydrogen bond and, thus, they are protected from exchange with protons of residual H_2_O in the organic solvent, whereas solvent exposed –OH protons exchange rapidly even if hydrogen bonded to the solvent [55]. 

**Figure 21:**
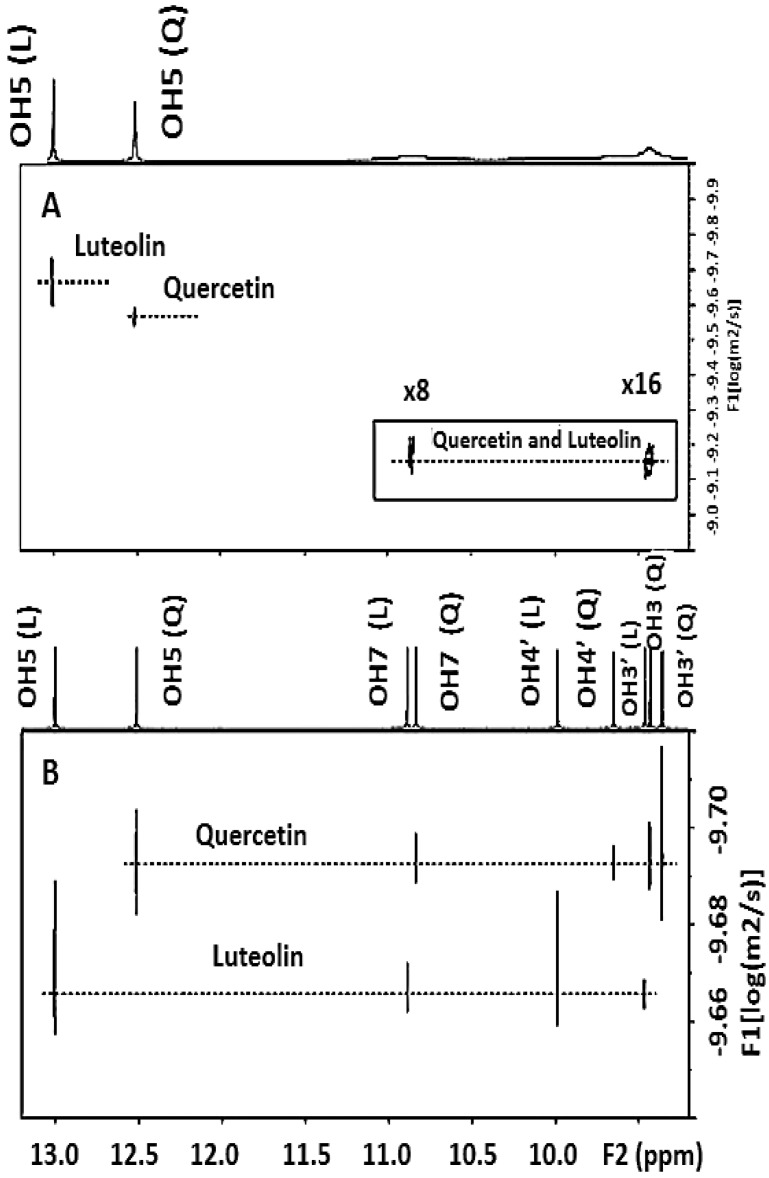
Fine-tuning of quercetin (Q) and luteolin (L) (Table 2) diffusion values. 500 MHz ^1^H-NMR DOSY of a mixture of luteolin (3.7 mM) and quercetin (3.5 mM) in DMSO-*d*_6_ (289 K, Δ = 100 ms, *δ*_g_ = 4.8 ms, ns = 16, total experimental time 27 min). The insert indicates the hydroxyl protons at a lower contour level (×8 and ×16). (**A**) Without and (**B**) with the addition of 0.07 mM picric acid (molar ratio [picric acid]/[luteolin]~0.014 and [picric acid]/[quercetin] = 0.015). Reproduced with permission from [55]. Copyright 2012, by Elsevier Science Ltd. (Amsterdam, The Netherlands).

Hong *et al.* [56] investigated the diffusion behavior of hydroxyl protons of quercetin in 100% DMSO-*d*_6_ and 90% DMSO-*d*_6_/10% H_2_O solutions with the use of ^1^H pulsed field gradient (PFG) NMR. The C-5 OH protons showed a biexponential diffusion decay due to exchange with H_2_O. The lifetime of the C-5 OH protons decreased from 96.7 ± 10.0 ms in 100% DMSO-*d*_6_ to 14.3 ± 1.4 ms in 90% DMSO-*d*_6_/10% H_2_O. It was concluded that PFG—NMR can provide a useful technique for investigating lifetimes of hydroxyl protons. A DOSY method also was developed for rapid identification of mixtures containing polyphenol organic compounds or crude reaction products. The method is based on the resolution enhancement of the resonances of the –OH protons and the fine—tuning of their diffusion coefficient in DMSO-*d*_6_ with the addition of picric acid and the use of temperatures near the freezing point of the solution (Figure 21) [55].

## 8. Deuterium Isotope Effects

*^n^Δ*C(OD) deuterium isotope effects have been extensively studied [3,12,57]. In cases that the OH(D) exchange rate is slow on the NMR time scale, two separate resonances are observed due to proton and deuterium species. The relative intensities will depend on the H:D fractionation ratio. *^n^Δ*OH(OD) long range isotope effects have also been observed in a number of molecules and their magnitude is larger in cases of strong hydrogen bonds. Thus, for the hypericin anion **4** (Figure 11) a positive isotope effect of the *peri* hydroxyl groups of 19 ppm has been observed which was related to the torsional deformation of the molecule [58].

## 9. Applications to Natural Products

Analysis of plant extracts is based on quite time-consuming chromatographic techniques and/or the use of specialized hyphenated NMR and MS techniques [59,60,61,62]. In the past few years, however, significant effort has been given to the development of NMR methods that can be applied to the analysis of complex plant extracts without separation or isolation of the individual compounds [63,64,65,66,67]. The use of ultra-high resolution in the phenolic –OH ^1^H-NMR spectroscopic region can provide a general method for the unequivocal structure analysis of natural products in complex plant extracts [16,17,68], in crude enzymatic transformations [55,69] and in the determination of total phenolics [70] and H_2_O_2_ [68,71] in complex mixtures. 

### 9.1. Mixture Analysis

**Figure 22:**
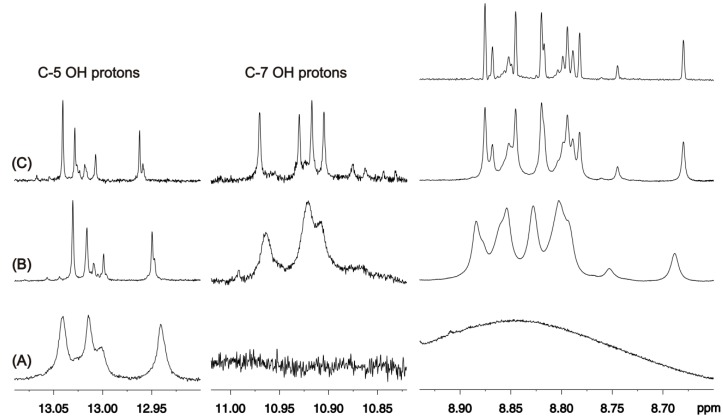
Selected regions of the 500 MHz 1D ^1^H-NMR spectra of 20 mg of an olive leaf methanol extract in 0.6 mL of DMSO-*d*_6_ (T = 288 K): (**A**) without the addition of picric acid; (**B**) with a mass ratio of [picric acid]/[extract] of 49.3 × 10^−3^; (**C**) the same solution as in (**A**) with a dilution factor of 8 and mass ratio of [picric acid]/[extract] of 49.4 × 10^−3^; upper trace, the same spectrum as in (**C**) with the application of Lorentz-to-Gauss transformation. Reproduced with permission from [16]. Copyright 2011, by the American Chemical Society.

Figure 22A illustrates selected regions of the ^1^H-NMR spectrum of the olive leaf methanol extract in DMSO-*d*_6_. The deshielded resonances in the region above 12.5 ppm, with line-widths ranging from 10.0 to 5.0 Hz, were attributed to the C-5 OH protons of flavonoids. No resonances were observed in the region *δ* = 10.5–11.5 ppm, where the C-7 OH groups of flavonoids are expected to give rise to ^1^H-NMR signals. In the region *δ* = 8.0–9.0 ppm (Figure 22A), a strong and broad resonance (Δ*ν*_1/2_ ≈ 250 Hz) was observed, which was attributed to oleuropein 6-*O*-*β*-d-glucopyranoside and its derivatives, as the main constituents of the extract [16]. Addition of picric acid (Figure 22B) in combination with dilution (Figure 22C) resulted in excellent resolution of all the hydroxyl group resonances. More specifically, three major peaks at *δ* = 13.03 ppm (Δ*ν*_1/2_ ≈ 0.7 Hz), 13.02 ppm (Δ*ν*_1/2_ ≈ 0.7 Hz), and 12.95 ppm (Δ*ν*_1/2_ ≈ 0.6 Hz) and several minor peaks were observed (Figure 21C). In the shielding region of the C-7 hydroxyl groups, four major peaks at *δ*= 10.97 ppm (Δ*ν*_1/2_ ≈ 1.1 Hz), 10.93 ppm (Δ*ν*_1/2_ ≈ 1.1 Hz), 10.92 ppm (Δ*ν*_1/2_ ≈ 1.1 Hz), and 10.91 ppm (Δ*ν*_1/2_ ≈ 1.1 Hz) were seen (Figure 22C). It should be emphasized that the addition of picric acid has a minor effect on ^1^H-NMR chemical shifts, which was found to be below 0.03 ppm; therefore, the ^1^H shieldings of the phenolic –OH groups can be of high diagnostic value for identifying individual components in complex plant extracts.

**Figure 23:**
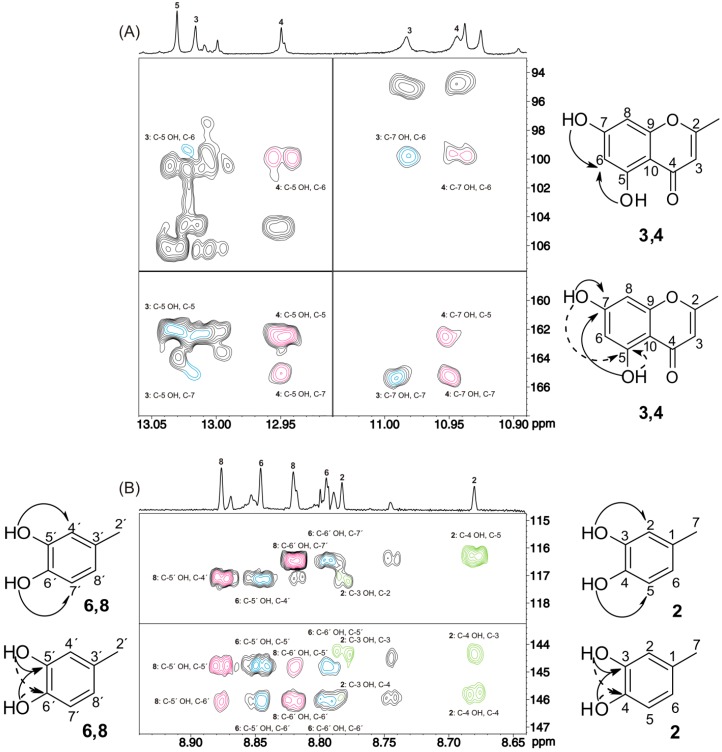
500 MHz 2D ^1^H-^13^C HMBC-NMR spectrum of 10 mg of an olive leaf methanol extract in 0.6 mL of DMSO-*d*_6_ with a mass ratio of [picric acid]/[extract] of 49.3 × 10^−3^ (T = 288 K, number of scans = 88, experimental time = 11 h and 34 min). The experiment was optimized for *^n^J*(^1^H, ^13^C) values of 6 to 8 Hz. (**A**) The common cross-peaks of the C-5 and C-7 hydroxyl protons to carbons C-6, C-5, and C-7 of luteolin-4′-*O*-*β*-d-glucopyranoside (**4**) and the common cross-peaks of the C-5 and C-7 hydroxyl protons to carbons C-6 and C-7 of luteolin (**3**) are illustrated in red and blue, respectively. (**B**) The common cross-peaks of the C-5′ and C-6′ hydroxyl protons to carbons C-4′ and C-7′, respectively (upper trace), and the common cross-peaks to carbons C-5′ and C-6′ (lower trace) are illustrated in blue for oleuropein 6-*O*-*β*-d-glucopyranoside (**6**), green for hydroxytyrosol (**2**), and red for oleuropein (aldehyde form) (**8**). Reproduced with permission from [16]. Copyright 2011, by the American Chemical Society.

**Figure 24:**
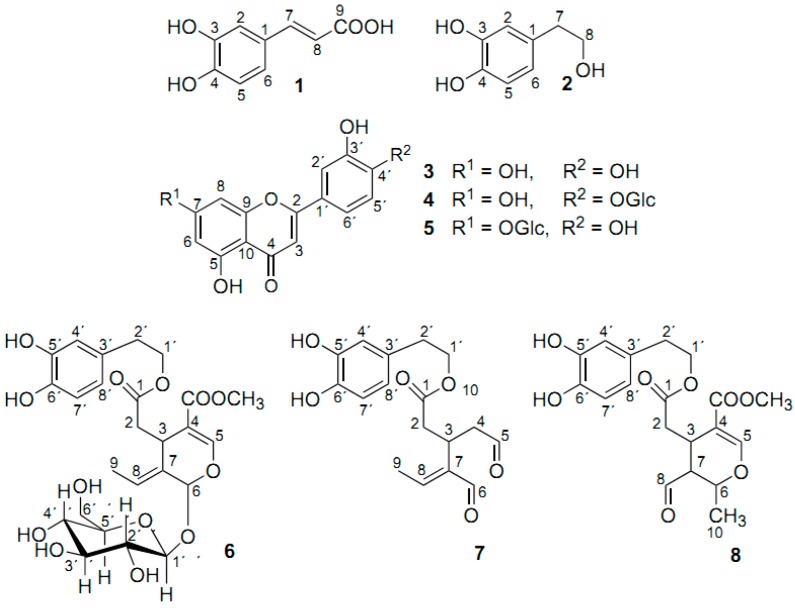
Chemical structures of the compounds which were identified in olive leaf methanol and aqueous extracts [16].

Application of the 2D ^1^H-^13^C HMBC method (Figure 23A) resulted in a significant number of *^n^J*(^1^H,^13^C) cross-peaks of the C-5 and C-7 hydroxyl groups, which allowed the assignments of compounds **3**–**4** (Figure 24). For example, the common cross-peaks of the C-5 and C-7 OH groups to C-6 (99.92 ppm), C-5 (162.55 ppm), and C-7 (165.05 ppm) of luteolin-4′-*O*-*β*-d-glucopyranoside (**4**) and the common cross-peaks of the C-5 and C-7 OH groups to C-6 (99.42 ppm) and C-7 (165.01 ppm) of luteolin (**3**) are of diagnostic value (Figure 23A).

**Scheme 2:**
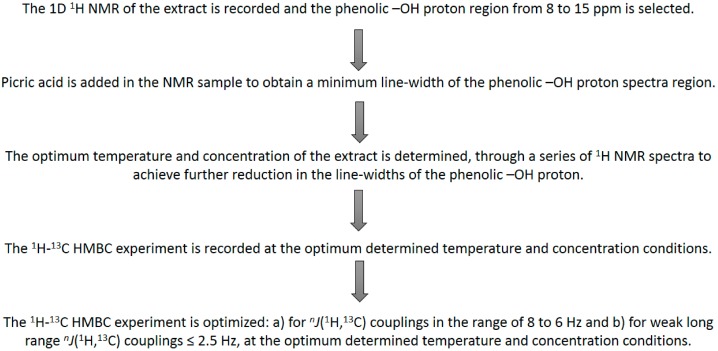
Experimental protocol for sequential and carbon resonance assignments [16].

Excellent resolution was also obtained in the hydroxyl group region *δ* = 8.6–8.9 ppm of oleuropein 6-*O*-*β*-d-glucopyranoside (**6**) and its derivatives. Application of the 2D ^1^H-^13^C HMBC pulse sequences (Figure 23B) showed a significant number of cross-peaks of the OH groups that allowed the assignment of the carbon skeletal of compounds **2**, **6**, and **8**. The resonances at *δ* = 8.66 and 8.77 ppm were attributed to the C-4 and C-3 hydroxyl protons, respectively, of hydroxytyrosol (**2**), since the cross-peaks to carbons at *δ* = 116.28, 144.37, and 145.83 and 117.22, 144.37, and 145.83 ppm, respectively, corresponded to those of the model compound. In addition, the C-4 and C-3 hydroxyl protons exhibited common cross-peaks to the C-4 and C-3 signals at *δ* = 144.37 and 145.83 ppm, respectively, which are of particular diagnostic value (Figure 23B). Similarly, the resonances at *δ* = 8.80 and 8.85 ppm and those at *δ* = 8.82 and 8.88 ppm were assigned to the C-6′ and C-5′ hydroxy protons of oleuropein 6-*O*-*β*-d-glucopyranoside (**6**) and oleuropein (aldehyde form) (**8**), respectively. The quantitative results for compounds **2-8 **are provided in Table 6 for methanol and aqueous extracts of olive leafs. The experimental protocol for sequential –OH and carbon skeletal resonance assignment in natural products is presented in Scheme 2. 

**Table 6:** Concentration levels *^a,b^* of compounds **2**–**8** of Scheme 3 in olive leaf extracts [16].

Extract	2	3	4	5	6	7	8
Methanol	7.0 ± 0.4	1.6 ± 0.1	2.9 ± 0.3	3.4 ± 0.2	36.9 ± 3.0	ND *^c^*	32.9 ± 2.0
Aqueous	1.3 ± 0.1	ND *^c^*	0.2 ± 0.05	0.4 ± 0.05	9.2 ± 1.1	7.2 ± 0.2	ND *^c^*

*^a^* mg g^−1^ extract. *^b^* Mean value of three measurements on three different extracts of the same sample ± S.D. (standard deviation). *^c^* ND = not detected.

**Figure 25:**
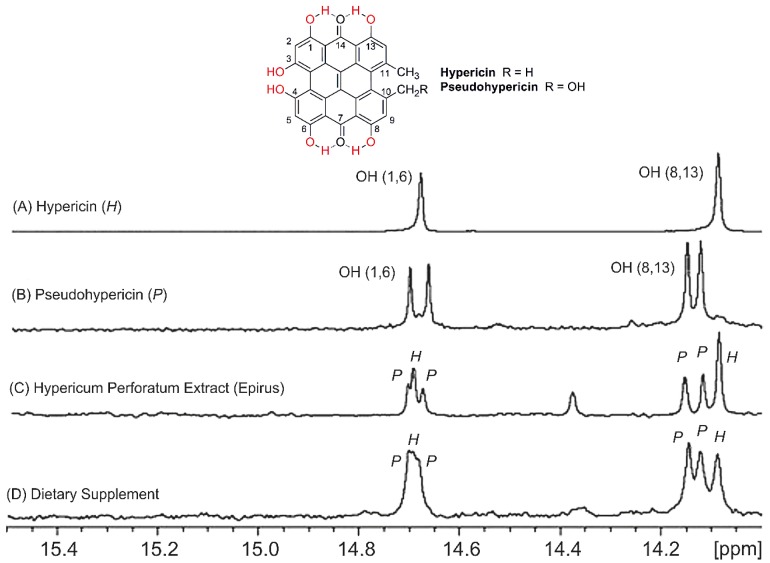
Selected region of 400 MHz ^1^H-NMR spectra of (**A**) hypericin; (**B**) pseudohypericin; (**C**) *Hypericum* *perforatum* extract with plant material from Epirus, Greece, and (**D**) dietary supplement. Number of scans: 1,024, acquisition time: 1.02 s, total experimental time: 102.7 min. P and H denote pseudohypericin and hypericin, respectively. Reproduced with permission from [72]. Copyright 2008, by Elsevier Science Ltd. (Amsterdam, The Netherlands).

The use of 2D ^1^H-^13^C HMBC experiments is not feasible for low concentration metabolites. However, even in these cases identification and quantification can be achieved provided that the –OH resonances of the minor metabolites are in a very characteristic chemical shift region. Figure 25 illustrates selected spectral region of the ^1^H-NMR spectrum of the model compounds hypericin and pseudohypericin and of four *H. perforatum* extracts. Identification and quantification of hypericin and pseudohypericin can be achieved in the characteristic region of 14–15 ppm [72,73]. The limits of detection (for S/N = 3) were found to be 2.8 μM for hypericin and 3.2 μM of pseudohypericin.

### 9.2. Determination of Total Phenolics

A novel method for the determination of total phenolic content was developed using ^1^H-NMR spectroscopy in the –OH spectral region of 8 to 15 ppm [70]. The determination was based on the following protocol:

(i) a 1D ^1^H-NMR spectrum was obtained in DMSO-*d*_6_;
(ii) a subsequent ^1^H-NMR spectrum was recorded with irradiation of the residual H_2_O resonance and
(iii) ^1^H-NMR spectra were recorded with the addition of a progressively increased amount of NaHCO_3_ salt.

Integration of the signal resonances between 15 to 8 ppm in spectrum (i) which were eliminated or reduced in intensity in steps (ii) and (iii), allowed the quantification of the total phenolic content. The results were expressed in mg of caffeic acid equivalence per gram of sample after proper data treatment.

The proposed ^1^H-NMR method for the determination of the total phenolic content has several advantages: (i) The sample pretreatment is minimal; (ii) no chemical reaction is needed in order to determine the phenolic content, with the exception of the need for partial neutralization of possible coexisting phenolic acids and (iii) the method is rapid since, for instance, at a concentration level of ~1 mmol L^−1^ of a phenolic compound would require ~10 min (3 spectra of one minute each, *n* = 3), whereas a spectrophotometric method, such as the FC method, or a chromatographic method would require approx. 1 hour [70]. Furthermore, a reaction factor *A*_e_ was proposed. Its value is an indicator of a compound reactivity (for *A*_e_ > 1 the reactive hydroxyl groups are less than the total hydroxyl groups); moreover the factor *A*_e_ allows the estimation of the availability of the hydroxyls of a matrix to participate in the redox reaction with the FC reagent. This is of great importance when an unknown extract/sample is measured.

The method was successfully applied for the total phenolic content determination in selected model compounds, an artificial mixture of them and several crude extracts of natural products [70]. Table 7 shows the results obtained for the determination of the total phenolic content for several plant extracts. For the rosemary, oregano and *Ligustrum lucidum* ethyl acetate extracts, the FC values were smaller than those obtained with the ^1^H-NMR method (having *A*_e_ > 1). This can be attributed to the presence of compounds bearing either non-reacting phenolic hydroxyls (such as in the case of gallic acid) or less reacting than total phenolic hydroxyls (such as in the case of flavonoids). The proposed ^1^H-NMR method, can also be applied for monitoring purposes in a wide range of matrixes from crude plant extracts and food products to biological fluids. 

## 10. Conclusions and Future Perspectives

The use of phenol –OH ^1^H-NMR in hydrogen bonding and conformational studies in solution presents experimental challenges due to rapid proton chemical exchange and, thus, extensive line broadening. However, careful optimization of experimental parameters that influence proton exchange rates e.g., pH, temperature and nature of the solvent, results in slow proton exchange rates and, thus, very sharp OH peaks with line widths Δν_1/2_ ≤ 2 Hz in favorable cases. This allows the application of two dimensional heteronuclear ^1^H-^13^C HMBC-NMR experiment to reveal long range coupling constants of hydroxyl protons and, thus, the unequivocal structure analysis even in cases of complex matrices of polyphenol natural products. 

**Table 7:** Total phenolic content and *A_e_* factor as determined for the plant extracts (caffeic acid equivalence, CAE per gram of sample) by the use of the FC reagent method and the ^1^H-NMR method [72].

Sample		Total Phenolic Content/ mg CAE g^−1^		*A_e_* ^d^
	FC ^a^	FC_corr_^b^	^1^H-NMR ^c^	
Sage ^e^	82 ± 8	66	55	0.83
Rosemary ^e^	97 ± 9	80	135	1.69
Oregano	211 ± 10	197	303	1.54
*Ligustrum lucidum* (MeOH extract)	61 ± 4	40	35	0.88
*Ligustrum lucidum* (H_2_O extract)	65 ± 3	45	15	0.33
*Ligustrum lucidum* ^e^	98 ± 8	89	115	1.29

^a^ *p* = 0.95, *n* = 3; ^b^ Corrected according to the relative recoveries; ^c^ %RSD = 2%, *n* = 3; ^d^ The FC_corr_ values were used; ^e^ Ethyl acetate extracts.

**Figure 26:**
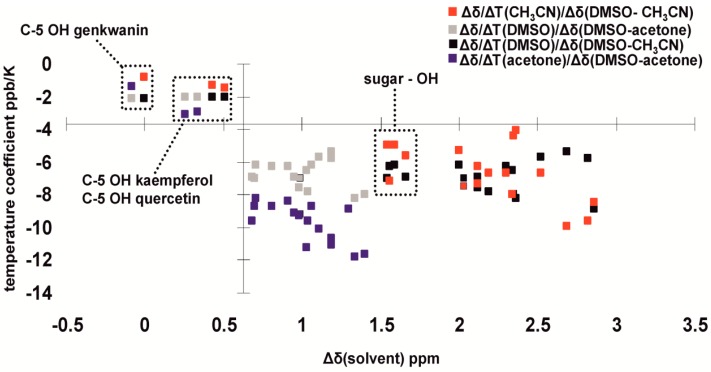
Correlation between the OH temperature coefficient and chemical shift differences in different solvents (DMSO-*d*_6_, acetone-*d*_6_ and CD_3_CN) of hydroxyl protons of oleuropein, hydroxytyrosol, quercetin, kaempferol and genkwanin. The C-5 OH protons of the flavonoids implicated in intramolecular hydrogen bonding are well clustered in the upper left corner of the graph. Reprinted with permission from [25]. Copyright 2013, The Royal Society of Chemistry.

Intramolecular and intermolecular hydrogen bonds have a very significant effect on ^1^H OH chemical shifts which cover a range from 4.5 ppm, for solvent exposed OH groups in low dielectric constant and hydrogen bonding ability solvents, up to 19 ppm in the case of extremely strong intramolecular hydrogen bonds such as in hypericin anions. Solvent effects on phenol –OH chemical shifts, temperature coefficients (Δ*δ*/ΔΤ), effects on OH diffusion coefficients and *^n^J*(^13^C, O^1^H) coupling constants can serve as reliable indicators of hydrogen bonding and solvation state of –OH groups (Figure 26). Since the clustering of the sugar OH groups appear in a specific location of the relevant graph, further research is needed with simple alcohols [74] and polyalcohols.

The range of ^1^H experimental chemical shifts of over 15 ppm due to (i) strong intramolecular and weak flip-flop intrarmolecular hydrogen bond interactions; (ii) solute-solvent intermolecular hydrogen bonds and (iii) conformational effects of substituents can be calculated accurately using a combination of DFT, polarizable continuum model (PCM) and discrete solute-solvent interaction with a single solvent molecule of each phenolic group. Excellent linear correlations between experimental and theoretical chemical shifts were obtained without using very large bases sets. The OH chemical shifts exhibit a strong linear dependence on the O(H)•••X hydrogen bond length of −8.86 ppm Å^−1^, however, further research is needed with compounds exhibiting very strong hydrogen bonds, such as in the case of hyperinate ionic forms. DFT calculations could also been performed to examine the conformational dependence of *^2^J*(^13^CO^1^H) and *^3^J*(^13^CCO^1^H) as in the case of exocyclic CO bonds in oligosaccharides [75].

The use of ultra-high resolution in the phenolic –OH ^1^H-NMR spectral region can provide a general method for the unequivocal structure analysis, qualitative and quantitative determination of phenol containing compounds and the determination of total phenolics in complex plant extracts without separation or isolation of the individual components. 
